# Pyridostigmine improves cardiac function and rhythmicity through RyR2 stabilization and inhibition of STIM1‐mediated calcium entry in heart failure

**DOI:** 10.1111/jcmm.16356

**Published:** 2021-03-23

**Authors:** Stephen Baine, Ingrid Bonilla, Andriy Belevych, Andrei Stepanov, Lisa E. Dorn, Radmila Terentyeva, Dmitry Terentyev, Federica Accornero, Cynthia A. Carnes, Sandor Gyorke

**Affiliations:** ^1^ College of Pharmacy The Ohio State University Columbus OH USA; ^2^ Department of Physiology and Cell Biology The Ohio State University Columbus OH USA

**Keywords:** autonomics, calcium, echocardiography, excitation contraction coupling, heart failure, hypertrophy, phosphorylation, Pyridostigmine, RyR2, STIM1

## Abstract

Heart failure (HF) is characterized by asymmetrical autonomic balance. Treatments to restore parasympathetic activity in human heart failure trials have shown beneficial effects. However, mechanisms of parasympathetic‐mediated improvement in cardiac function remain unclear. The present study examined the effects and underpinning mechanisms of chronic treatment with the cholinesterase inhibitor, pyridostigmine (PYR), in pressure overload HF induced by transverse aortic constriction (TAC) in mice. TAC mice exhibited characteristic adverse structural (left ventricular hypertrophy) and functional remodelling (reduced ejection fraction, altered myocyte calcium (Ca) handling, increased arrhythmogenesis) with enhanced predisposition to arrhythmogenic aberrant sarcoplasmic reticulum (SR) Ca release, cardiac ryanodine receptor (RyR2) hyper‐phosphorylation and up‐regulated store‐operated Ca entry (SOCE). PYR treatment resulted in improved cardiac contractile performance and rhythmic activity relative to untreated TAC mice. Chronic PYR treatment inhibited altered intracellular Ca handling by alleviating aberrant Ca release and diminishing pathologically enhanced SOCE in TAC myocytes. At the molecular level, these PYR‐induced changes in Ca handling were associated with reductions of pathologically enhanced phosphorylation of RyR2 serine‐2814 and STIM1 expression in HF myocytes. These results suggest that chronic cholinergic augmentation alleviates HF via normalization of both canonical RyR2‐mediated SR Ca release and non‐canonical hypertrophic Ca signaling via STIM1‐dependent SOCE.

## 1 INTRODUCTION

Heart failure (HF) is a significant cause of mortality and morbidity in the United States. With the ageing population, HF incidence is expected to increase over time.^1^ Autonomic imbalance is a key component of the pathophysiology of HF.^2^ Following a decrement in cardiac output, a compensatory increase in sympathetic outflow results in increased norepinephrine release, which acutely improves ventricular contractility and heart rate to maintain cardiac output. Over time, however, chronic sympathetic stimulation leads to maladaptive cardiac remodelling. Conversely, parasympathetic activity is withdrawn in patients with HF,^3^ which results in decreased heart rate variability (HRV) and baroreflex sensitivity (BRS) that are correlated with increased mortality.^4^,^5^

Treatments to restore autonomic balance by increasing parasympathetic outflow have shown utility improving HF morbidity and mortality, although the results have not been consistent.^6^, ^7^, ^8^, ^9^, ^10^, ^11^, ^12^, ^13^, ^14^, ^15^, ^16^ Indeed, vagal nerve stimulation has been shown to increase survival in post‐MI rats and improve autonomic balance in dogs with HF.^6^, ^7^, ^8^ In humans, evidence from initial clinical trials ^9^, ^10^, ^11^ suggested that vagal nerve stimulation (VNS) may be a promising treatment for patients with HF via improvement in ejection fraction and reduced end‐diastolic volume. On the other hand, pharmacological agents that increase acetylcholine levels in the neuro‐effector junction would be predicted to increase cholinergic transmission similar to vagal nerve stimulation. Pyridostigmine (PYR), an FDA‐approved acetylcholinesterase inhibitor, prevents degradation of acetylcholine (ACh) thus increasing ACh concentration in the synaptic cleft. Lataro et al^12^ demonstrated improved cardiac performance associated with increased VEGF production following chronic PYR administration in post‐MI rats. Moreover, in human HF, PYR was shown to prevent premature ventricular complexes, improve heart rate recovery following exercise and improve short‐term HRV.^13^, ^14^, ^15^ Thus, a developing line of evidence implicates PYR as a potentially non‐invasive therapeutic option for cardiovascular disease.^12^, ^29^ However, the mechanisms of PYR treatment and therapeutic efficacy in HF remain to be determined.

Alterations of calcium (Ca) release via cardiac ryanodine receptors (RyR2) are thought to contribute to several key pathologies in HF including hypertrophy, arrhythmogenesis and reduced contractility.^30^ Dysfunctional RyR2s in HF have been linked to altered sympathetic regulation associated with β‐AR‐dependent stimulation of CaMKII with subsequent hyper‐phosphorylation of RyR2 at serine 2814.^31^, ^32^ More recently, hypertrophy, HF and arrhythmia have been linked to up‐regulation of store‐operated Ca entry (SOCE).^33^, ^34^ SOCE occurs when lowering of luminal Ca prompts the SR protein, STIM1, to actuate Ca entry through plasmalemmal Ca channels.^35^ SOCE, considered to be most prevalent in non‐excitable cells, has been shown to operate in diseased cardiomyocytes in parallel with RyR2‐mediated Ca signaling.^36^ However, the relative roles of these Ca signaling mechanisms in cardiac disease and in the beneficial effects of muscarinic stimulation remain to be elucidated.

The current study investigates chronic PYR treatment in a transverse aortic constriction (TAC) model of HF in mice. We hypothesize that chronic PYR treatment ameliorates HF‐related abnormalities in Ca handling thereby attenuating HF development. The beneficial effects of PYR may be mediated through hampering RyR2‐mediated SR Ca release and/or STIM1‐mediated SOCE. In order to test this hypothesis, we examined the effects of PYR treatment on intracellular calcium dynamics, cardiac structural remodelling, contractile performance and arrhythmia vulnerability in vivo.

## 2 METHODS

All animal procedures were approved by The Ohio State University Institutional Animal Care and Use Committee and conformed to the Guide for the Care and Use of Laboratory Animals published by the US National Institute of Health (NIH Publication No. 85‐23, revised 2011).

### 2.1 Transverse aortic constriction

Approximately 50 age‐matched (2 month) male C57BL/6J mice (Wild‐Type, WT, Jackson Labs #000664) were anaesthetized with 2% isoflurane and intubated for artificial ventilation at 120‐160 breaths per minute, of 0.2‐0.35 mL. Heating pads were used to keep body temperature at 37°C throughout the procedure. The transverse aorta was accessed via a left lateral thoracotomy and 6‐0 suture used to ligate the aorta overlying a blunted 25‐ or 27‐gauge needle. Mice were recovered on a heating pad and assessed for cardiac dysfunction using echocardiography.

### 2.2 Osmotic pump implantation

Mice subjected to TAC surgery recovered for 7 days prior to osmotic pump implantation. Experimental groups were defined as CTL (Control, no TAC surgery, no osmotic pump), TAC (TAC surgery, implanted with 0.9% saline osmotic pump) and TAC + PYR (TAC surgery, implanted with 2‐10 mg/kg pyridostigmine bromide). (Figure S1A) Prior to implantation, osmotic pumps (Alzet model 1004) were filled with pyridostigmine bromide (PYR) or sterile 0.9% saline. Pumps were primed by incubation in sterile 0.9% saline at 37°C for 30 minutes prior to insertion. Mice were anaesthetized with 2% isoflurane, and osmotic pumps were implanted subcutaneously above right hind limb. PYR was supplied at range of 2‐10 mg/kg/day for 28 days at a volume of 0.11 µL/hr.

### 2.3 Cardiomyocyte isolation

Intact ventricular myocytes were obtained by enzymatic digestion as previously described.^37^ Briefly, mice were anaesthetized with 5% isoflurane in 95% oxygen until a deep plane of anaesthesia was achieved. Hearts were rapidly excised and cannulated through the aorta for perfusion with ice‐cold calcium‐free Tyrode's solution containing (in mM) 140 NaCl, 5.4 KCl, 0.5 MgCl_2_, 10 HEPES and 5.5 glucose with pH 7.4. Cannulated hearts were then switched to a gravity flow Langendorff apparatus containing calcium‐free Tyrode's solution with a temperature of 37˚C. Hearts were perfused for 5 minutes before switching to a perfusion solution containing Liberase TH (0.24 U; Roche) for digestion of connective tissue. Following enzymatic digestion, hearts were minced and triturated in perfusion solution containing BSA (20 mg/mL).

### 2.4 Echocardiography

Echocardiographic analysis was performed on mice anaesthetized with isoflurane (1.5% in 1 L/min oxygen). Mice were immobilized on a heated imaging stage during image acquisition. Long‐ and short‐axis analyses were conducted using the GE LOGIQ E ultrasound machine. Analysis was conducted (M‐Mode) following acquisition using at least three non‐adjacent contractions. Operators were blinded to experimental group.

### 2.5 Electrocardiography

Electrocardiography (ECG) recordings were performed before and after epinephrine and caffeine challenge as previously described.^38^ Briefly, ECG recordings were obtained from mice anaesthetized with isoflurane (1‐1.5%). Subcutaneous electrodes were placed in the left, right upper and right lower limbs for ECG recording (PL3504 PowerLab 4/35, ADInstruments). After a baseline recording (5 minutes), a stress challenge was performed by administering an intraperitoneal injection with epinephrine (Epi, 1.5 mg/kg) and caffeine (Caff, 120 mg/kg). ECG recording continued for 15 minutes after challenge. Analysis was performed using the LabChart 7.3 program (ADInstruments). Ventricular arrhythmias were defined as frequent ectopies, bigeminies and/or ventricular tachycardia (VT).

### 2.6 Acetylcholinesterase assay

Blood samples (80‐150 µL) were collected sublingually from mice in tubes containing 3% heparin. Blood was centrifuged at 8000 RPM for 4‐5 minutes at room temperature, and plasma was collected. Acetylcholinesterase activity was detected using Abcam acetylcholinesterase assay kit (Abcam) following manufacturer's instructions. Plasma AChE activity was detected as absorbance change (410 ± 5 nm) using a Fisher Scientific MultiSkan FC spectrophotometer.

### 2.7 Calcium imaging

Ventricular myocyte cytoplasmic Ca was recorded as described previously.^39^ Myocytes were plated on 12 mm coverslips covered by laminin (50 mg/mL). Cells were incubated with a Ca‐sensitive dye Fluo‐4 AM (9 μM, Thermo Fisher Scientific) in a low Ca (0.4 mM CaCl_2_) external solution at room temperature for 20‐25 minutes. Following 15‐20 minutes of dye washout, myocytes were continuously perfused with a solution containing (in mM) 140 NaCl, 5.4 KCl, 2.0 CaCl_2_, 0.5 MgCl_2_, 5.6 glucose and 10 HEPES (pH 7.4). Ca transients were elicited by field stimulation using SD9 stimulator (Grass Technologies/Astro‐Med Inc.). Intracellular Ca imaging was performed using line‐scanning mode of Olympus FluoView FV 1000 (Olympus America Inc.) confocal microscope system equipped with 60x oil‐immersion objective lens (NA 1.4). Fluo‐4 was excited with 488 nm line of argon laser, and signal was collected at 500‐600 nm wavelengths. Following background (non‐cellular signal) subtraction, spatially averaged fluorescence profiles were normalized to the baseline cellular fluorescence (F0).

The propensity of ventricular myocytes to diastolic Ca waves was assessed in the presence of 100 nM isoproterenol (ISO), a β‐adrenergic receptor agonist, at 1 Hz stimulation. The average delay between electrical stimulus and the onset of Ca wave in the following diastolic interval was calculated, and time dependence of cumulative probability of Ca waves was created using *survival* and *survminer* packages of R software (R Foundation for Statistical Computing, http://www. R‐project.org).

### 2.8 Western blot

Isolated ventricular myocytes and/or ventricular tissue were digested in RIPA Buffer (Sigma) with protease and phosphatase inhibitors (Sigma). Protein concentrations were determined by Bradford assay. Cardiac homogenates (25‐50 µg) were subjected to 4%‐15% SDS PAGE (Bio‐Rad) and blotted onto nitrocellulose membranes (Bio‐Rad). Phosphorylation status of proteins was detected using phospho‐specific and total protein antibodies including RyR2‐Ser‐2814 (Badrilla A010‐31AP), RyR Total (Thermo Fisher #MA3‐916), CaMKII‐Thr‐287 (PA5‐37833), total CaMKII (Cell Signaling 3362S) and GAPDH (Fitzgerald #G109a). The ratio between phospho/total protein was obtained for values. For STIM1 (Sigma, #S6072) and Orai1 (Alomone Labs, #ALM‐025), expression was normalized to GAPDH levels. GAPDH was used as a loading control. Images were processed with ImageJ software (NIH).

### 2.9 mRNA expression analysis

RNA was extracted from whole heart tissue using TRIzol (Invitrogen 15596026), and reverse transcription was performed using the High Capacity cDNA Reverse Transcription Kit (Applied Biosystems 4368814). Selected genes were analysed by real‐time polymerase chain reaction using SYBR green (Bio‐Rad 1725272). Quantified mRNA expression was normalized to Rpl7 (ribosomal protein L7) and expressed relative to controls. Primers used were as follows: *MYH6* 5′‐ GGAGGTGGAGAAGCTGGAA 3′‐ ATCTTGCCCTCCTCATGCT; *MYH7* 5′‐ CACCAACAACCCCTACGATT 3′‐ AGCACATCAAAGGCGCTATC; *NPPA* 5′‐ CACAGATCTGATGGATTTCAAGA 3′‐ CCTCATCTTCTACCGGCATC; and *NPPB* 5′‐ GTCAGTCGCTTGGGCTGT 3′‐ CAGAGCTGGGGAAAGAAG.

### 2.10 Store‐operated calcium entry (SOCE)

SOCE events were measured following previously published methods.^36^ Briefly, cardiac myocytes were loaded with Fluo‐4 AM for 20 minutes in 0.5 mM Ca external solution containing 140 NaCl, 5.4 KCl, 0.5 mM MgCl_2_, 10 HEPES and 5.5 glucose pH 7.4 at room temperature. Following initial dye loading, Ca depletion solution (0 Ca Tyrode and (in µM): 500 caffeine, 2 thapsigargin (TG), 10 verapamil and 1 SEA0400) was added to the cells and incubated at room temperature (RT) for 10‐20 minutes to deplete myocyte SR Ca (TG and caffeine), while inhibiting voltage‐dependent Ca channels (verapamil) and Na+/Ca2 + exchange (SEA0400). In order to observe SOCE signals, a solution containing 2 mM Ca Tyrode and (in µM): 2 thapsigargin, 10 verapamil and 1 SEA0400 was rapidly applied during confocal Ca imaging protocol.

### 2.11 Statistical analysis

Statistical analyses were completed using Origin and/or Microsoft Excel. Unpaired one‐tailed Student's *t* test or 1‐way analysis of variance (ANOVA) with post hoc Fisher's test or Tukey HSD was used to test statistical significance between experimental groups. Outlier data points were excluded by using the Grubbs outlier test with significance level of Alpha 0.05.

## 3 RESULTS

**FIGURE 1:**
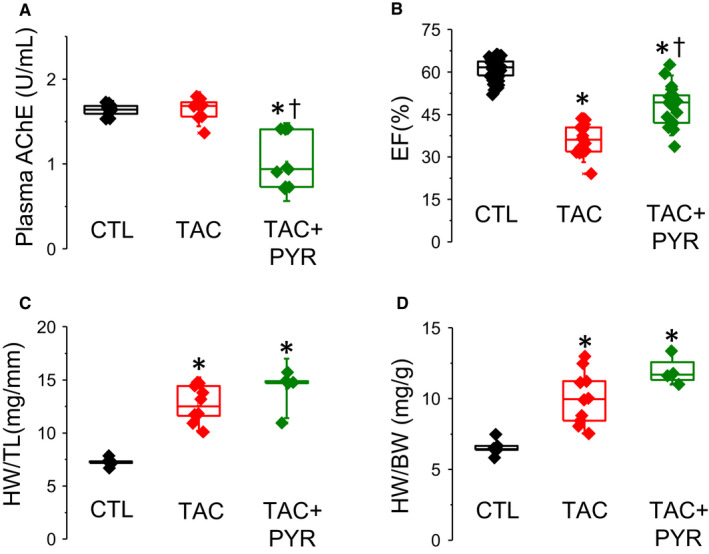
Pyridostigmine improves in vivo cardiac function following TAC surgery but does not prevent TAC‐induced structural remodelling of the heart. (A) Plot of acetylcholinesterase activity 28 days after osmotic pump implantation (*P < 0.05 vs. CTL, †*P* < 0.05 vs. TAC), 1‐way ANOVA + Tukey HSD. For acetylcholinesterase activity, n = 10 mice per group. (B) Plot of ventricular ejection fraction (EF, expressed as a %) TAC resulted in a significant reduction in EF vs. CTL mice (*P < 0.000001 vs. CTL). However, chronic pyridostigmine treatment significantly increased EF vs. TAC mice (†*P* < 0.0001 vs. TAC), 1‐way ANOVA + Tukey HSD. For echocardiographic measurements, analysis was conducted following acquisition using at least three non‐adjacent contractions. (C) Plot of heart weight (HW) to tibia length (TL) ratio. TAC mice exhibited a significant increase in HW/TL ratio vs. CTL mice (*P < 0.0001 vs. CTL) and chronic pyridostigmine treatment failed to reduce HW/TL following TAC (*P < 0.0001 vs. CTL), 1‐way ANOVA + Tukey HSD. (D) Plot of heart weight (HW) to bodyweight (BW) ratio. TAC hearts show a significant increase in HW/BW vs. CTL hearts (*P < 0.0001 vs. CTL), and pyridostigmine failed to reduce HW/BW ratio in response to TAC (*P < 0.0001 vs. CTL), 1‐way ANOVA + Tukey HSD. For hypertrophy measurements, n = 7‐10 mice per group. For all plots, mean ± SD of data indicated by line and a minimum of three experiments per group

We investigated the effects of chronic treatment with the acetylcholinesterase inhibitor, pyridostigmine bromide (PYR), on in vivo cardiac function and myocyte Ca handling in TAC mice. For chronic PYR treatment, 7 days after TAC surgery mice were implanted with osmotic pumps delivering the drug (at 0.11 µL/hr) for 28 days (Figure S1A). To determine in vivo inhibition of acetylcholinesterase, plasma was separated from blood following 28 days of osmotic pump treatment. As shown in Figure 1A, chronic PYR treatment resulted in a significant inhibition of plasma acetylcholinesterase activity compared to CTL and TAC samples at 28 days post‐implantation.

### 3.1 PYR improves ventricular function in TAC mice

Echocardiographic analysis was conducted to determine in vivo ventricular function and hypertrophy following TAC surgery (Figure 1B and Figure S1B). A significant decrease in ventricular function measured by ejection fraction (EF) was observed in untreated TAC mice consistent with pressure overload–induced HF (Figure 1B). Interestingly, chronic PYR treatment significantly increased EF compared to TAC mice, although the values of these parameters stayed below CTL levels (Figure 1B). Consistent with previous studies,^40^ EF in TAC mice showed substantial variability, although with no obvious batch‐dependent correlation between the TAC and PYR groups (Figure S1C). Echocardiographic analysis also showed increased interventricular septal thickness at end‐diastole and end‐systole (IVSd/IVSs) in TAC hearts indicating cardiac hypertrophy (Table S2). PYR treatment resulted in a significant, albeit incomplete improvement in IVSd in TAC hearts (Table S2). Structural remodelling was further assessed by measuring the heart weight/body weight (HW/BW) and heart weight/tibial length (HW/TL) in CTL, TAC and TAC + PYR mice (Figure 1C,D). TAC mice exhibited increased HW/TL and HW/BW relative to the CTL group, but these parameters were not significantly different from the chronic PYR treatment group (Figure 1C,D).

**FIGURE 2:**
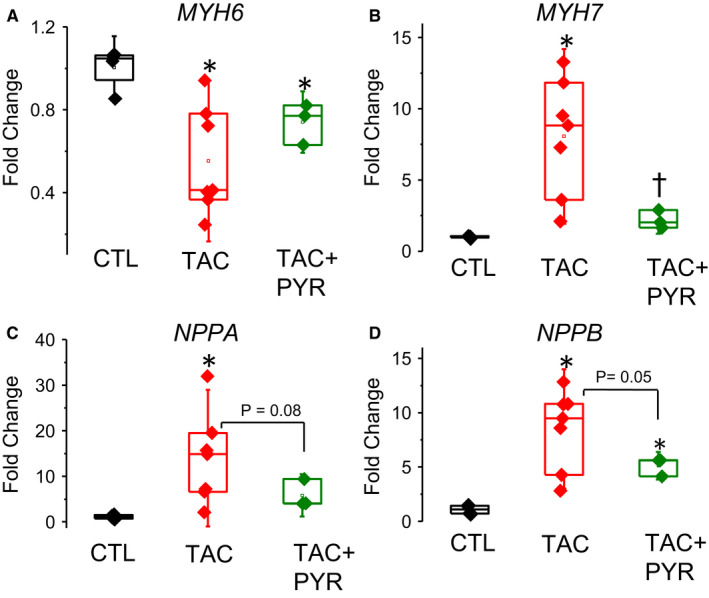
Pyridostigmine reduces foetal gene expression in TAC mice. (A) Representative plot of MYH6 (myosin heavy chain 6) mRNA expression. TAC induced a significant decrease in adult MYH6 mRNA expression vs. CTL hearts (*P < 0.05 vs. CTL), while pyridostigmine did not attenuate MYH6 mRNA expression vs. CTL hearts (*P < 0.05 vs. CTL), 1‐way ANOVA + Tukey HSD. (B) Representative plot of MYH7 (myosin heavy chain 7) mRNA expression. TAC hearts exhibited a significant increase in MYH7 mRNA expression vs. CTL hearts (*P < 0.05 vs. CTL), while pyridostigmine significantly reduced MYH7 expression following TAC (*P < 0.05 vs. TAC), 1‐way ANOVA + Tukey HSD. (C) Representative plot of NPPA (atrial natriuretic peptide) mRNA expression. TAC hearts exhibit increased NPPA mRNA expression vs. CTL hearts (*P < 0.05 vs. CTL) and pyridostigmine treatment trends towards a decrease in NPPA expression (*P* = 0.08 vs. TAC), 1‐way ANOVA + Tukey HSD. (D) Representative plot of NPPB (brain natriuretic peptide) mRNA expression. TAC induced a significant increase in NPPB mRNA expression vs. CTL hearts (*P < 0.05 vs. CTL), while pyridostigmine trended towards a decrease in NPPB expression following TAC surgery (*P* = 0.05 vs. TAC), 1‐way ANOVA + Tukey HSD. For all plots, mean ± SD of data indicated by line. Data points are listed as fold change + standard deviation. For foetal gene expression, N = 3‐7 mice per group, minimum of 3 experiments per group

Additionally, we examined the impact of PYR treatment on induction of foetal genes as markers of pathological cardiac remodelling in TAC mice. As expected, HF in TAC mice was associated with an isoform switch from α‐MHC (α myosin heavy chain, MYH6) to foetal β‐MHC (β‐myosin heavy chain, MYH7) (Figure 2A,B) as well as increased expression of atrial natriuretic peptide (ANP, NPPA) and brain natriuretic peptide (BNP, NPPB) (Figure 2C,D). PYR treatment resulted in a partial normalization of MYH6 mRNA and nearly complete return to CTL values of MYH7, NPPA and NPPB mRNA levels (Figure 2A‐D). Collectively, these results suggest PYR treatment improves ventricular function and partially alleviates adverse ventricular remodelling in pressure overload–induced HF.

### 3.2 PYR reduces arrhythmia susceptibility in TAC mice

**FIGURE 3:**
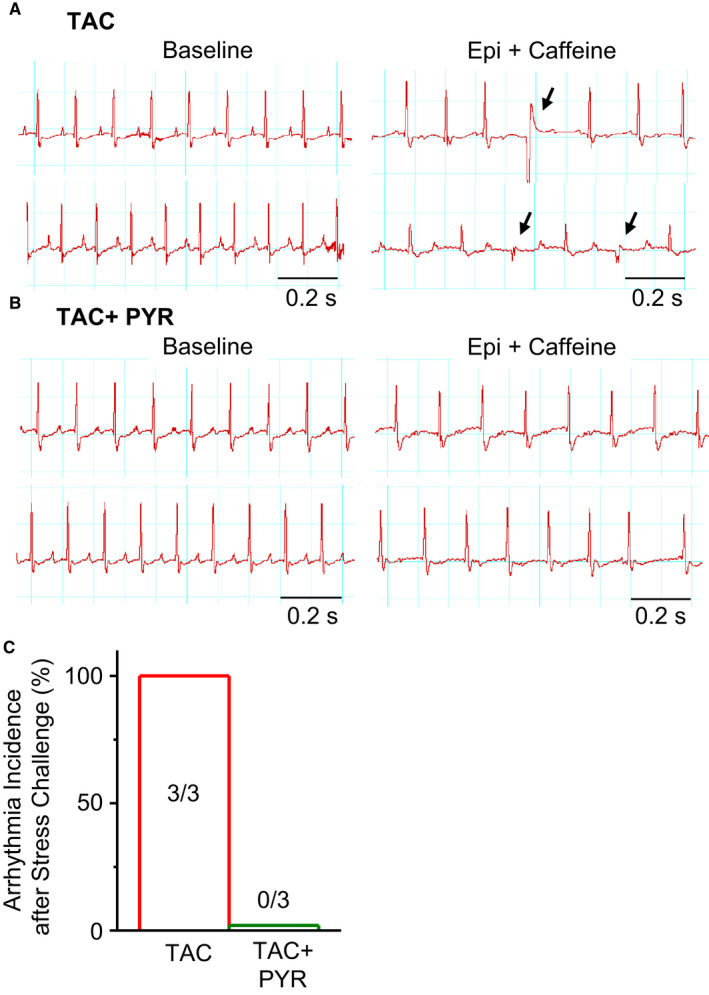
Pyridostigmine prevents cardiac arrhythmias in pressure overload HF. (A, B) Representative ECG traces obtained under baseline and 10‐min stress challenge with epinephrine (1.5 mg/kg) plus caffeine (120 mg/kg) in TAC (A) or pyridostigmine treated mice (B). Arrows indicate premature ventricular complex (PVC). Summary plots showing PVC frequency (C) and incidence of arrhythmias in TAC and TAC + PYR groups

Pressure overload HF is associated with increased risk of ventricular arrhythmia. Therefore, we performed ECG measurements to assess the effects of PYR on arrhythmia vulnerability in TAC mice. ECG measurements were performed in anaesthetized CTL, TAC and TAC + PYR mice challenged with epinephrine and caffeine. In congruence with previous reports, untreated TAC mice exhibited enhanced predisposition to arrhythmogenesis indicated by frequent premature ventricular contractions (PVCs) (Figures 3A,C) Notably, the stress challenge failed to induce PVCs in the TAC + PYR group (Figure 3B‐C). Taken together, these data suggest that PYR treatment confers protection against arrhythmia in pressure overload HF.

### 3.3 PYR improves myocytes Ca handling in TAC myocytes

**FIGURE 4:**
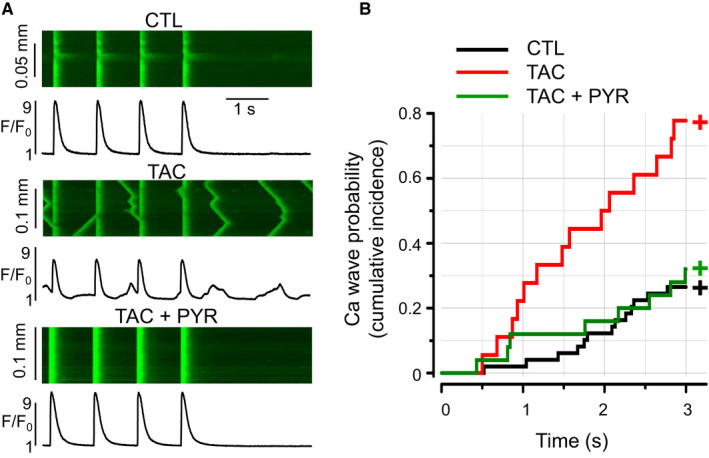
Pyridostigmine reduces frequency of arrhythmogenic calcium waves in pressure overload–induced HF. (A) Representative confocal line‐scan images along with spatially averaged fluorescence profiles recorded in myocytes from CTL, TAC and TAC + PYR groups, respectively. Cells were stimulated at 1 Hz in a presence of 100 nM isoproterenol, a beta‐adrenergic receptor agonist. (B) Summary plot of time dependence of calcium wave probability. TAC myocytes showed significant increase in calcium wave incidence (*P* < 0.0001, CTL *vs*. TAC, log‐rank test). Pyridostigmine treatment resulted in a significant reduction of calcium waves in TAC myocytes (*P* < 0.02, TAC *vs*. TAC + PYR, log‐rank test). Data were obtained from CTL (*h* = 5, n = 49), TAC (*h *= 2, n = 18) and TAC + PYR (*h* = 4, n = 25) groups, where *h* is the number of cell isolations, and *n* is the number of myocytes studied

Aberrant SR Ca release in the form of spontaneous cytosolic Ca waves is a characteristic feature of HF and an established cause of both impaired cardiac contractile function and arrhythmogenesis, particularly under conditions of catecholamine stress.^41^ Therefore, we performed measurements of cytosolic Ca in isolated myocytes derived from CTL, TAC and TAC + PYR mice to assess the effect of chronic treatment with PYR on myocyte Ca handling in HF under baseline conditions and in the presence of ISO (100 nM) (Figure 4). Under baseline conditions, Ca transients were similar in control and TAC myocytes (Table S1). Chronic PYR treatment had no effect on Ca transient amplitude, while decreasing the decay rate of the Ca transients in TAC myocytes (Table S1). SR Ca content in the three experimental groups was assessed through application of caffeine (10 mM). We found no significant alterations in the SR Ca content in TAC and TAC + PYR myocytes relative to control and TAC, respectively (Figure S2). Exposure to ISO (100 nM) markedly increased Ca transient amplitude and decay rate in control, TAC and TAC + PYR myocytes at all pacing rates (Table S1). The stimulatory effect of ISO on Ca transient amplitude was most pronounced in TAC + PYR myocytes at 2 Hz. Under baseline conditions, TAC + PYR myocytes exhibited slowed Ca transients relative to both control and TAC myocytes. However, in the presence of ISO, myocytes from the three groups showed similar Ca transient decay rates. As expected, in the presence of ISO, TAC myocytes displayed increased predisposition to arrhythmogenic Ca waves relative to control (Figure 4). Notably, chronic treatment with PYR reduced the incidence of arrhythmogenic calcium waves in TAC myocytes. (Figure 4) Overall, our studies indicate PYR reduces aberrant calcium release and confers protection against arrhythmia in pressure overload HF.

### 3.4 PYR improves dysregulated CAMKII‐RyR2 S2814 signaling

**FIGURE 5:**
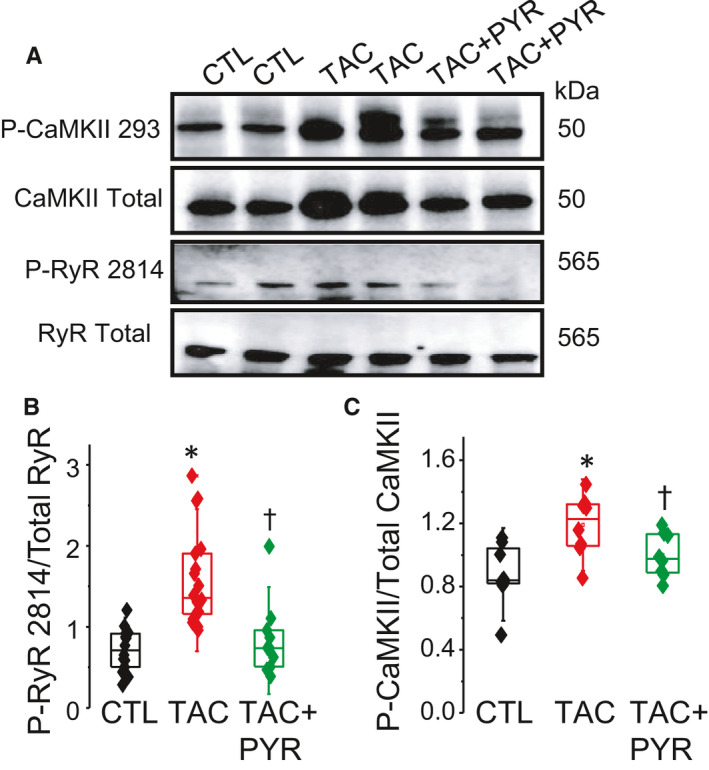
Pyridostigmine reduces TAC‐mediated increase in CAMKII activity. (A) Representative Western blot showing total and phosphorylated levels of CaMKII and RYR2. (B) and (C) Boxplots illustrating the effect of pyridostigmine treatment on TAC‐induced increase in RyR2 S2814 phosphorylation (*, *P* < 0.01 vs. CTL, †, *P* < 0.05 vs. TAC) and in CaMKII T287 phosphorylation (*P < 0.05 *vs*. CTL; †*P* < 0.05 *vs*. TAC). 1‐way ANOVA + Tukey HSD, n = 3‐5 mice per group, and minimum of three experiments per group

**FIGURE 6:**
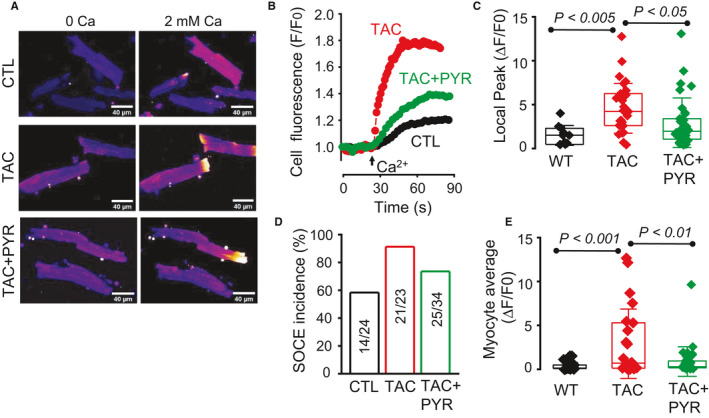
Pyridostigmine reduces STIM1‐dependent store‐operated Ca entry (SOCE) in HF myocytes. (A) Representative images of Fluo‐4 fluorescence illustrating SOCE response in cardiac myocytes from CTL, TAC and TAC + PYR groups. Myocyte sarcoplasmic reticulum (SR) Ca was depleted by 1 µM thapsigargin, a SR Ca ATPase inhibitor, and SOCE was induced by rapid increase in extracellular Ca from 0 to 2 mM. (B) Representative time‐courses of spatially averaged fluorescence before and following application of Ca were recorded in myocytes from CTL, TAC, and TAC + PYR groups. (C) Summary data on the amplitude of local Ca increase in CTL, TAC and TAC + PYR myocytes, 1‐way ANOVA + Tukey HSD. (D) Summary data for the fraction of cells exhibiting SOCE response in CTL, TAC and TAC + PYR groups, respectively. (E) Boxplot illustrating maximal changes in myocyte fluorescence in response to application of external Ca, 1‐way ANOVA + Tukey HSD. For SOCE experiments, n = 11‐34 cells per group, n = 3‐10 mice per group, minimum of three experiments

Aberrant SR Ca release in HF has been associated with increased phosphorylation of RyR2 at serine 2814 (S2814) by CaMKII in both human and animal models.^31^, ^32^ We examined the effects of PYR treatment on RyR2 CaMKII phosphorylation in TAC hearts using immunoblot assays. In TAC hearts, Western blot analyses revealed a significant increase in RyR2 S2814 phosphorylation, consistent with previous reports (Figure 5A,B). Notably, chronic PYR treatment resulted in a significant reduction in RyR2 S2814 phosphorylation compared to untreated TAC hearts (Figure 5A,B). Additionally, we investigated the upstream signaling effector CAMKII to determine the link between PYR treatment and decreased RyR2 S2814 phosphorylation. As shown in Figure 6A and C, TAC resulted in a significant increase in CaMKII activation level indexed by CAMKII phosphorylation at T287. Notably, PYR treatment blunted the increase in CaMKII‐T287 phosphorylation following TAC surgery. Taken together, our results indicate a mechanistic link for PYR protection in response to pressure overload by preventing dysregulation of SR Ca release via reduced CaMKII phosphorylation of RyR2 S2814.

### 3.5 PYR reduces STIM1‐dependent SOCE in HF myocytes

**FIGURE 7:**
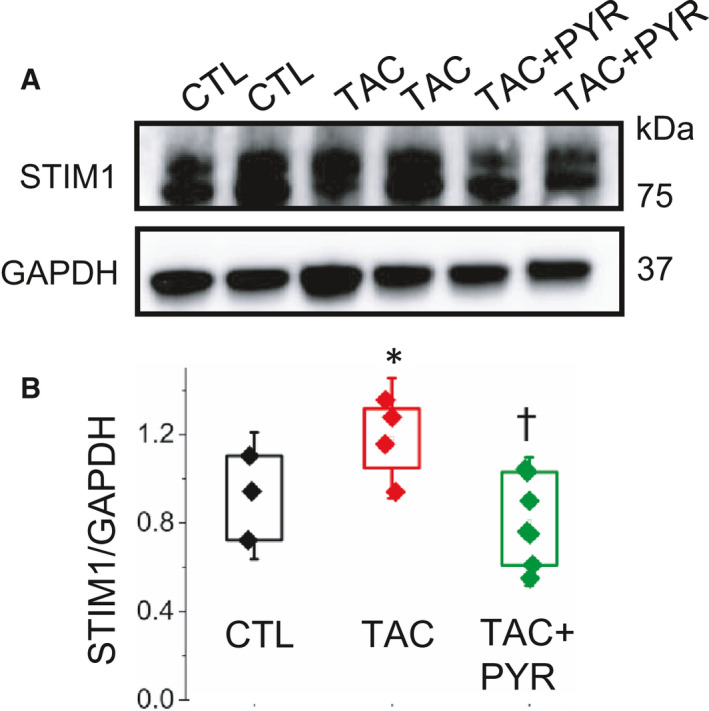
Pyridostigmine effects STIM1 levels in control, HF and HF + PYR myocytes. (A) Representative Western blot showing STIM1 levels detected in heart preparations from CTL, TAC and TAC + PYR groups, respectively. GAPDH was used as a loading control. (B) Boxplot illustrating the effect of pyridostigmine treatment on TAC‐induced increase in STIM1 levels (*P < 0.05 *vs*. CTL, †*P* < 0.05 *vs*. TAC, 1‐way ANOVA + Tukey HSD, n = 3‐4 mice per group, minimum of three experiments per group

Up‐regulated STIM1‐dependent Ca signaling has been implicated in pathologic hypertrophy, HF and arrhythmogenesis.^34^, ^42^ We investigated the effects of PYR treatment on SOCE and its effectors, STIM1 and Orai1, in TAC myocytes. We performed measurements of SOCE in the form of local Ca entry events (LoCEs,^36^) in CTL, TAC and TAC + PYR myocytes. As recently reported, LoCEs represent Ca entry via store‐operated Ca entry sites predominantly localized at myocyte intercalated discs (IDs).^36^ In accordance with our previous report,^36^ LoCEs occurred mainly at myocyte ends (Figure 6A). LoCEs magnitude and incidence were significantly increased in TAC myocytes compared with CTL cells (Figure 6A‐E). Notably, PYR significantly reduced SOCE in TAC myocytes (Figure 6D‐E). To assess whether the observed changes in SOCE with PYR treatment were associated with similar changes with STIM1 and ORAI1 expression, we measured protein levels with Western blot. STIM1 levels were significantly increased in TAC compared to both CTL and TAC + PYR groups (Figure 7A and B). ORAI1 expression trended higher in TAC vs. CTL groups and reduced in TAC + PYR groups vs. TAC groups, and the data showed no statistically significant differences in ORAI1 levels between the three groups (Figure S3). Collectively, these results suggest that the beneficial effects of PYR on TAC hearts are associated with down‐regulation of STIM1‐mediated Ca signalling.

## 4 DISCUSSION

The current study examined the impact and underlying mechanisms of chronic treatment with the cholinesterase inhibitor, pyridostigmine (PYR), in pressure overload HF induced by aortic constriction (TAC). PYR treatment arm showed better contractile performance and rhythmic activity than that of untreated TAC mice. At the same time, PYR improved altered intracellular Ca handling by inhibiting aberrant SR Ca release and diminishing pathologically enhanced SOCE in HF myocytes. At the molecular level, these PYR‐induced changes in Ca handling were associated with reductions of pathologically enhanced phosphorylation of RyR2 S2814 and expression of STIM1 in HF myocytes. These results suggest that chronic cholinergic augmentation alleviates HF via normalization of both canonical RyR2‐mediated SR Ca release and non‐canonical hypertrophic Ca signaling via STIM1‐dependent SOCE.

Accumulating evidence suggests that parasympathetic augmentation through inhibition of AChE with PYR may provide a non‐invasive therapeutic option for cardiovascular disease.^12^, ^29^ In particular, PYR has been reported to reduce arrhythmogenesis in patients while improving cardiac performance and decreasing fibrosis^13^, ^14^, ^15^in post‐MI rats.^12^ However, its therapeutic efficacy and mechanisms of action in HF of different aetiologies remain to be elucidated. Our results showed that PYR alleviates adverse functional alterations and partially attenuates structural remodelling in pressure overload‐induced HF. Moreover, our results suggest PYR modulates RyR2‐mediated Ca signaling via decreasing RyR2 S2814 phosphorylation and normalization of STIM1‐governed SOCE.

RyR2 hyperactivity has been proposed as an important factor in pathophysiology of HF. A large body of experimental evidence suggests that enhanced RyR2 phosphorylation by CaMKII (at Ser‐2814) results in aberrant SR Ca release via RyR2s that manifests itself as both diastolic SR Ca leak and arrhythmogenic Ca waves.^31^, ^32^ Activation of CaMKII is in turn attributable to increased cytosolic Ca and cyclic adenosine monophosphate(cAMP)/exchange protein directly activated by cyclic AMP 2 (cAMP/EPAC2) signaling^43^ as well as elevated levels of reactive oxygen species (ROS) in the setting of chronic sympathetic overstimulation during HF development.^44^, ^45^ Stimulation of the parasympathetic branch might be expected to reverse these detrimental effects of the sympathetic system on myocyte Ca handling. Indeed, our present study show, for the first time, that the beneficial effects of PYR on cardiac contractile performance and rhythmicity in HF are associated with normalization of RyR2 Ser‐2814 phosphorylation and myocyte Ca cycling. These findings are consistent with our previous results demonstrating that muscarinic stimulation with CCh reduces CaMKII phosphorylation of RyR2 S2814 in canine HF myocytes.^46^ Reduced RyR2 2814 phosphorylation could be attributed to the reversal of stimulation of the cAMP/EPAC/CaMKII pathway upon stimulation of muscarinic receptor 2 (MR2).^43^ Another possibility could involve inhibition of ROS‐dependent stimulation of CAMKII upon activation of muscarinic receptor 3 (MR3).^46^Further studies are needed to define the specific molecular steps that mediate reduced RyR2 CaMKII phosphorylation by PYR in different cardiac disease settings.

Recently, it has been shown that up‐regulated STIM1‐governed SOCE plays a critical role in cardiac hypertrophy and arrhythmogenesis.^47^ Our results revealed that parasympathetic augmentation by PYR alleviates arrhythmogenesis and hypertrophy while suppressing up‐regulation of SOCE and STIM1 in HF. We found no significant differences in ORAI1 in TAC or PYR treated myocytes. These results are consistent with the finding that up‐regulation of SOCE in cardiac disease may involve increased complexation of STIM1 and ORAI1 or increased expression of these proteins.^36^ Up‐regulation of STIM1 in HF appears to occur as part of induction of the foetal genes via Ca‐dependent activation of the calcineurin/nuclear factor activator of T cells (NFAT) pathway.^34^ Consistent with this notion, up‐regulation of STIM1 in HF was associated with up‐regulation of key foetal genes MYH6, MYH7, NPPA and NPPB (Figure 2). Moreover, normalization of SOCE and STIM1 in HF myocytes by PYR was accompanied by normalization of expression of these foetal genes (ie MYH6, MYH7 and NPPB) (Figure 2).

Given our finding that PYR affects both RyR2 and SOCE signaling (Figures 5 and 6), it is important to consider the primary site of action of muscarinic augmentation by PYR in HF. Despite being localized to different subcellular microdomains, T‐tubules and intercalated discs respectively,^36^ these systems appear to influence each other activities and show an ability to operate in an integrated manner. A CaMKII phosphorylation‐dependent increase in RyR2 activity could enhance SOCE through increasing fractional SR Ca release and the degree of SR Ca depletion following SR Ca release.^48^ Over time, CaMKII‐mediated RyR2 activity could also increase SOCE by facilitating STIM1 expression as part of the induction of the foetal gene programme (Figure 6). At the same time, increased SOCE could result in further increases in CaMKII‐dependent RyR2 S2814 phosphorylation especially in myocyte regions near intercalated discs (ID) (where SOCE is primarily localized). Therefore, we propose that parasympathetic augmentation via PYR acts primarily on RyR2 function by reversing RyR2 CaMKII phosphorylation, alleviating SR Ca leak with subsequent reversal of STIM1 up‐regulation in failing myocytes. However, we cannot exclude more direct effects of muscarinic stimulation on SOCE in failing cardiomyocytes. Modulation of SOCE by muscarinic signaling awaits further investigation.

It is to be noted that although PYR reversed nearly completely certain parameters including EF, arrhythmia vulnerability, other parameters, particularly those associated with cardiac structural remodelling remained largely unaffected (eg HW/TL, HW/BW). These results are consistent with complex, heterogeneous cardiovascular effects of vagal nerve stimulation in cardiac disease.^49^, ^50^, ^51^ Additional studies with PYR and other AChE inhibitors at different concentrations are needed to further evaluate the factors underlying these heterogeneous effects of PYR define optimal conditions for their effective therapeutic use.

Given that the mouse TAC model is not a close approximation of most forms of human HF, it may not consistently correlate directly to HF patients. Additionally, the beneficial effects of parasympathetic augmentation including AChE inhibitor application may reportedly be mediated through different mechanisms including actions on oxidative, inflammatory and fibrotic processes in the heart and other systems. Nevertheless, altered myocyte Ca handling, owing to both abnormal SR Ca release via RyR2 hyper‐phosphorylated at S2814 and up‐regulated SOCE are recognized features of human HF.^32^, ^52^ Thus, our results regarding the effects of PYR on myocyte Ca handling in the mouse might be also relevant to human. Further studies are needed to define the prevailing mechanisms of the beneficial effects, and the therapeutic efficacy of AChE inhibitors is needed to support their use in patients with cardiac disease.

## CONFLICT OF INTEREST

The authors declare they have no conflicts of interest.

## AUTHOR CONTRIBUTIONS

**Stephen Baine:** Conceptualization (lead); Data curation (lead); Formal analysis (lead); Investigation (lead); Methodology (lead); Project administration (lead); Visualization (supporting); Writing‐original draft (lead); Writing‐review & editing (lead). **Ingrid Bonilla:** Data curation (supporting); Formal analysis (supporting); Investigation (supporting); Writing‐original draft (supporting); Writing‐review & editing (supporting). **Andriy Belevych:** Conceptualization (supporting); Data curation (supporting); Formal analysis (supporting); Investigation (supporting); Methodology (supporting); Writing‐review & editing (supporting). **Andrei Stepanov:** Data curation (supporting); Formal analysis (supporting); Investigation (supporting); Writing‐review & editing (supporting). **Lisa E. Dorn:** Data curation (supporting); Formal analysis (supporting); Investigation (supporting); Writing‐review & editing (supporting). **Radmila Terentyeva:** Data curation (supporting); Formal analysis (supporting); Investigation (supporting); Methodology (supporting); Writing‐original draft (supporting); Writing‐review & editing (supporting). **Dmitry Terentyev:** Formal analysis (supporting); Investigation (supporting); Project administration (supporting); Validation (supporting); Writing‐review & editing (supporting). **Federica Accornero:** Data curation (supporting); Formal analysis (supporting); Investigation (supporting); Project administration (supporting); Resources (supporting); Software (supporting); Supervision (supporting); Writing‐review & editing (supporting). **Cynthia A. Carnes:** Investigation (supporting); Methodology (supporting); Project administration (supporting); Resources (supporting); Software (supporting); Supervision (supporting); Validation (supporting); Visualization (supporting); Writing‐review & editing (supporting). **Sandor Gyorke:** Conceptualization (supporting); Funding acquisition (lead); Investigation (supporting); Methodology (supporting); Project administration (supporting); Resources (lead); Software (supporting); Supervision (lead); Validation (supporting); Visualization (supporting); Writing‐original draft (supporting); Writing‐review & editing (supporting).

## Supporting information

Supplementary Material

## Data Availability

All data are contained within the manuscript.
