# Supplementary material for: Pyridostigmine improves cardiac function and rhythmicity through RyR2 stabilization and inhibition of STIM1‐mediated calcium entry in heart failure
Source: J Cell Mol Med. 2021 Mar 23;25(10):4637–48. doi: 10.1111/jcmm.16356 (PMC8107086; doi:10.1111/jcmm.16356)
Supplement: Supplementary file 1 — Supplementary Material [file JCMM-25-4637-s001.pptx]

## Slide 1
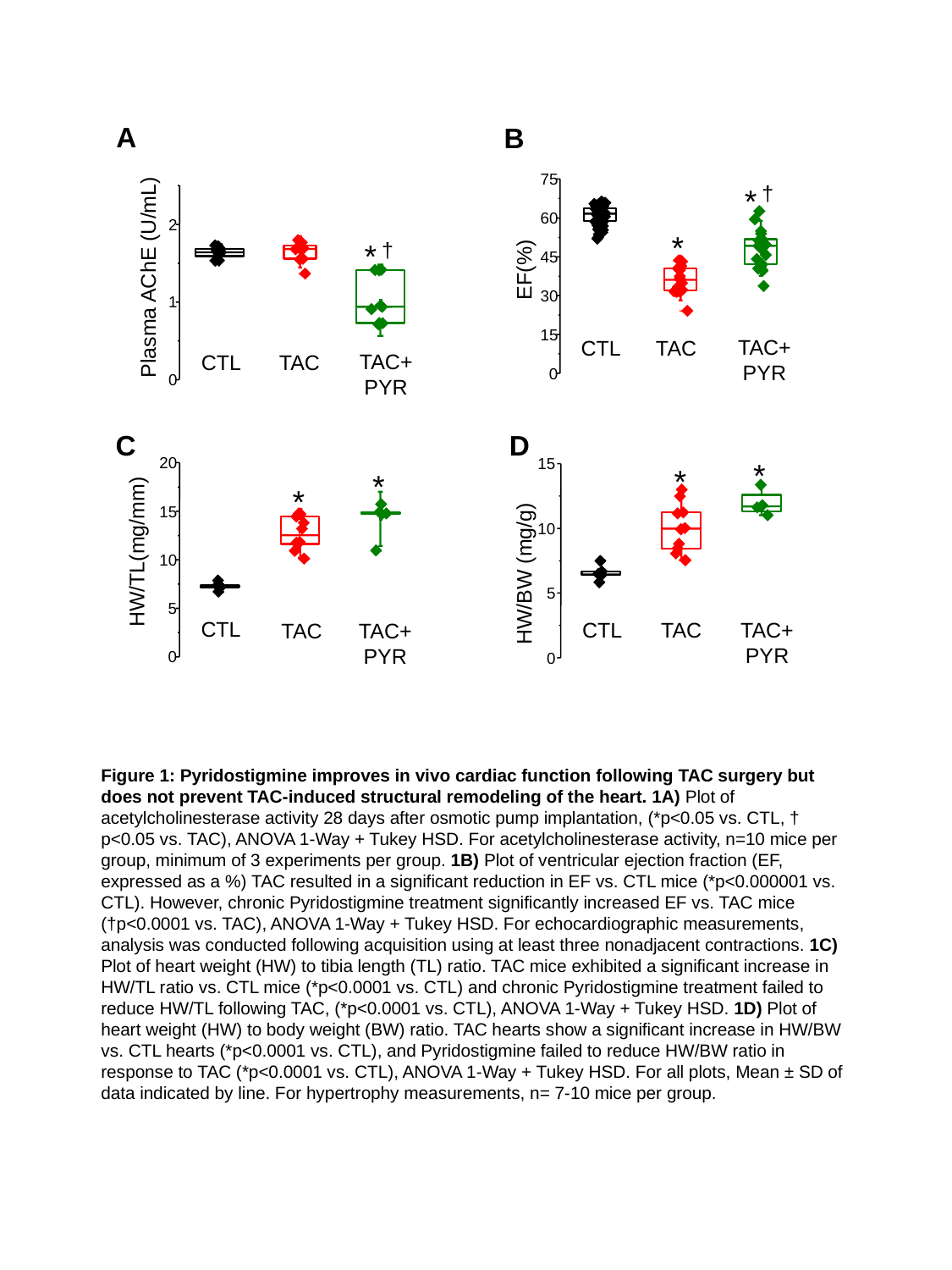

A
B
75
†
*
60
*
45
30
15
0
2
*
†
1
0
EF(%)
Plasma AChE (U/mL)
TAC+
PYR
CTL
TAC
TAC+
PYR
CTL
TAC
C
D
20
*
*
15
10
5
0
15
*
*
10
HW/TL(mg/mm)
HW/BW (mg/g)
5
CTL
CTL
TAC
TAC+
PYR
TAC+
PYR
TAC
0
Figure 1: Pyridostigmine improves in vivo cardiac function following TAC surgery but does not prevent TAC-induced structural remodeling of the heart. 1A) Plot of acetylcholinesterase activity 28 days after osmotic pump implantation, (*p<0.05 vs. CTL, † p<0.05 vs. TAC), ANOVA 1-Way + Tukey HSD. For acetylcholinesterase activity, n=10 mice per group, minimum of 3 experiments per group. 1B) Plot of ventricular ejection fraction (EF, expressed as a %) TAC resulted in a significant reduction in EF vs. CTL mice (*p<0.000001 vs. CTL). However, chronic Pyridostigmine treatment significantly increased EF vs. TAC mice (†p<0.0001 vs. TAC), ANOVA 1-Way + Tukey HSD. For echocardiographic measurements, analysis was conducted following acquisition using at least three nonadjacent contractions. 1C) Plot of heart weight (HW) to tibia length (TL) ratio. TAC mice exhibited a significant increase in HW/TL ratio vs. CTL mice (*p<0.0001 vs. CTL) and chronic Pyridostigmine treatment failed to reduce HW/TL following TAC, (*p<0.0001 vs. CTL), ANOVA 1-Way + Tukey HSD. 1D) Plot of heart weight (HW) to body weight (BW) ratio. TAC hearts show a significant increase in HW/BW vs. CTL hearts (*p<0.0001 vs. CTL), and Pyridostigmine failed to reduce HW/BW ratio in response to TAC (*p<0.0001 vs. CTL), ANOVA 1-Way + Tukey HSD. For all plots, Mean ± SD of data indicated by line. For hypertrophy measurements, n= 7-10 mice per group.

## Slide 2
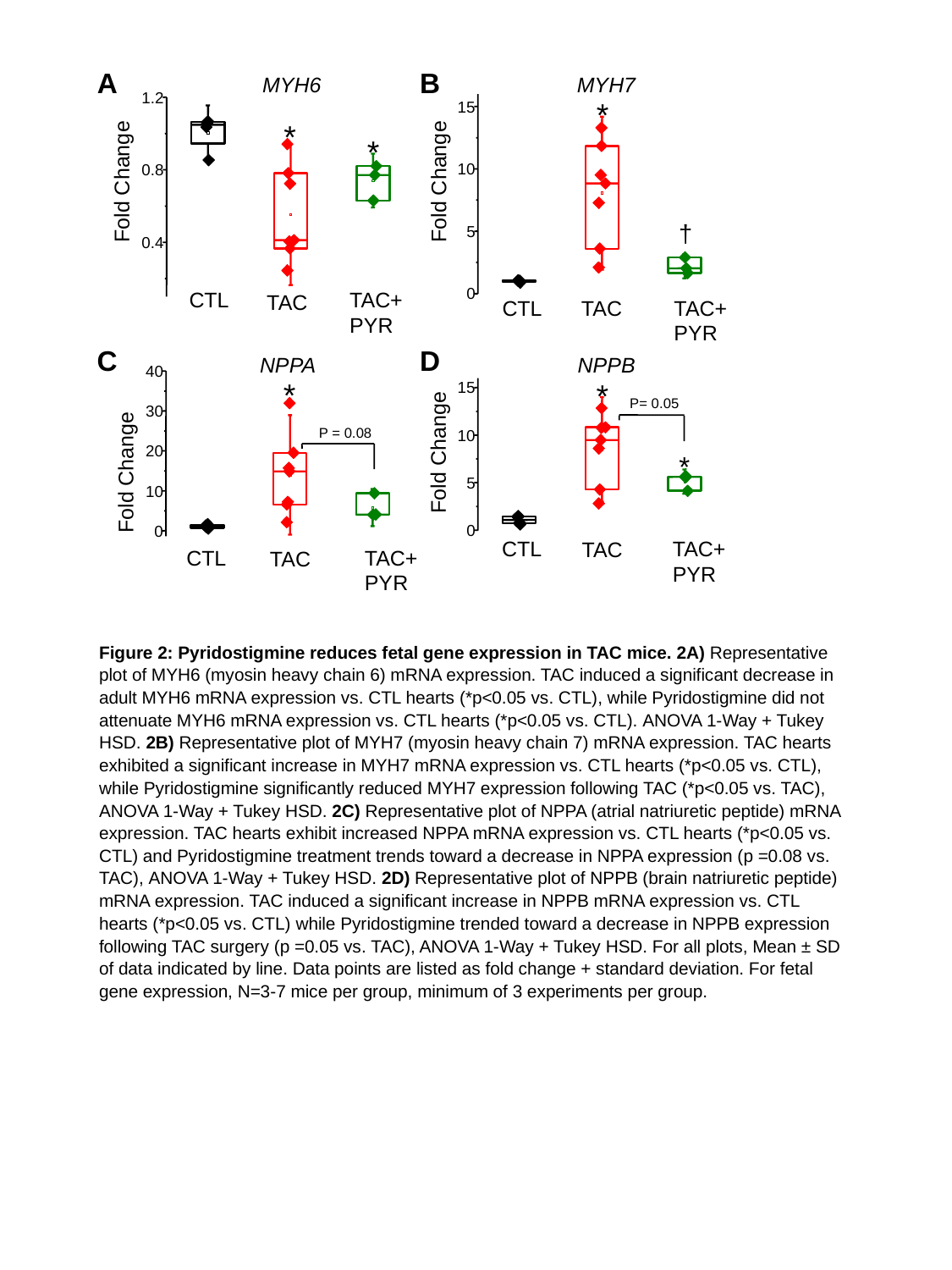

A
B
MYH6
MYH7
*
15
10
†
5
0
1.2
*
*
0.8
Fold Change
Fold Change
0.4
CTL
TAC+
PYR
TAC
CTL
TAC
TAC+
PYR
C
D
NPPA
NPPB
40
*
30
P = 0.08
20
10
0
*
15
P= 0.05
10
Fold Change
*
Fold Change
5
0
CTL
TAC+
PYR
TAC
CTL
TAC+
PYR
TAC
Figure 2: Pyridostigmine reduces fetal gene expression in TAC mice. 2A) Representative plot of MYH6 (myosin heavy chain 6) mRNA expression. TAC induced a significant decrease in adult MYH6 mRNA expression vs. CTL hearts (*p<0.05 vs. CTL), while Pyridostigmine did not attenuate MYH6 mRNA expression vs. CTL hearts (*p<0.05 vs. CTL). ANOVA 1-Way + Tukey HSD. 2B) Representative plot of MYH7 (myosin heavy chain 7) mRNA expression. TAC hearts exhibited a significant increase in MYH7 mRNA expression vs. CTL hearts (*p<0.05 vs. CTL), while Pyridostigmine significantly reduced MYH7 expression following TAC (*p<0.05 vs. TAC), ANOVA 1-Way + Tukey HSD. 2C) Representative plot of NPPA (atrial natriuretic peptide) mRNA expression. TAC hearts exhibit increased NPPA mRNA expression vs. CTL hearts (*p<0.05 vs. CTL) and Pyridostigmine treatment trends toward a decrease in NPPA expression (p =0.08 vs. TAC), ANOVA 1-Way + Tukey HSD. 2D) Representative plot of NPPB (brain natriuretic peptide) mRNA expression. TAC induced a significant increase in NPPB mRNA expression vs. CTL hearts (*p<0.05 vs. CTL) while Pyridostigmine trended toward a decrease in NPPB expression following TAC surgery (p =0.05 vs. TAC), ANOVA 1-Way + Tukey HSD. For all plots, Mean ± SD of data indicated by line. Data points are listed as fold change + standard deviation. For fetal gene expression, N=3-7 mice per group, minimum of 3 experiments per group.

## Slide 3
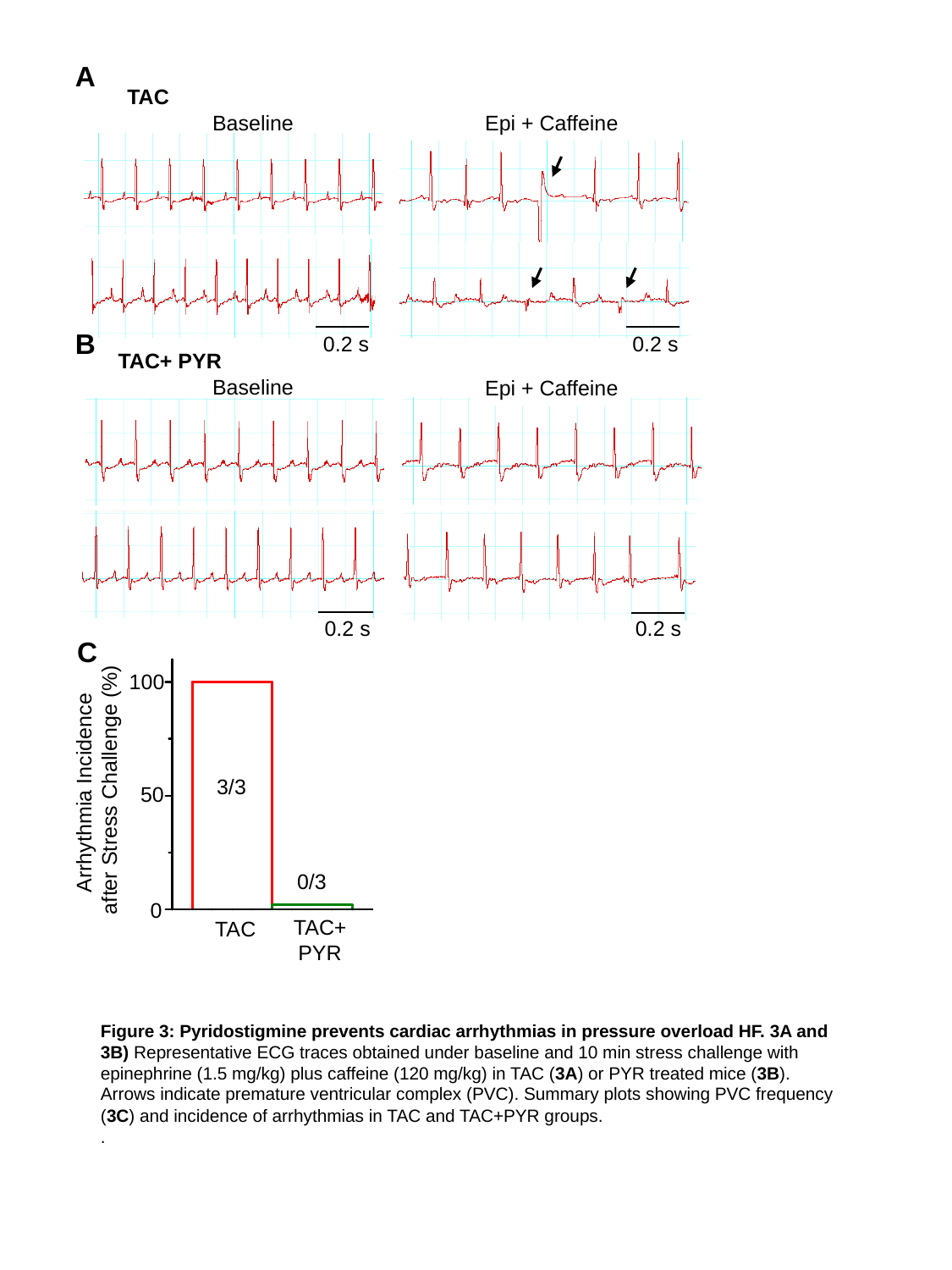

A
TAC
Baseline
Epi + Caffeine
B
0.2 s
0.2 s
TAC+ PYR
Baseline
Epi + Caffeine
0.2 s
0.2 s
C
100
Arrhythmia Incidence
 after Stress Challenge (%)
3/3
50
0/3
0
TAC+
PYR
TAC
Figure 3: Pyridostigmine prevents cardiac arrhythmias in pressure overload HF. 3A and 3B) Representative ECG traces obtained under baseline and 10 min stress challenge with epinephrine (1.5 mg/kg) plus caffeine (120 mg/kg) in TAC (3A) or PYR treated mice (3B). Arrows indicate premature ventricular complex (PVC). Summary plots showing PVC frequency (3C) and incidence of arrhythmias in TAC and TAC+PYR groups.
.

## Slide 4
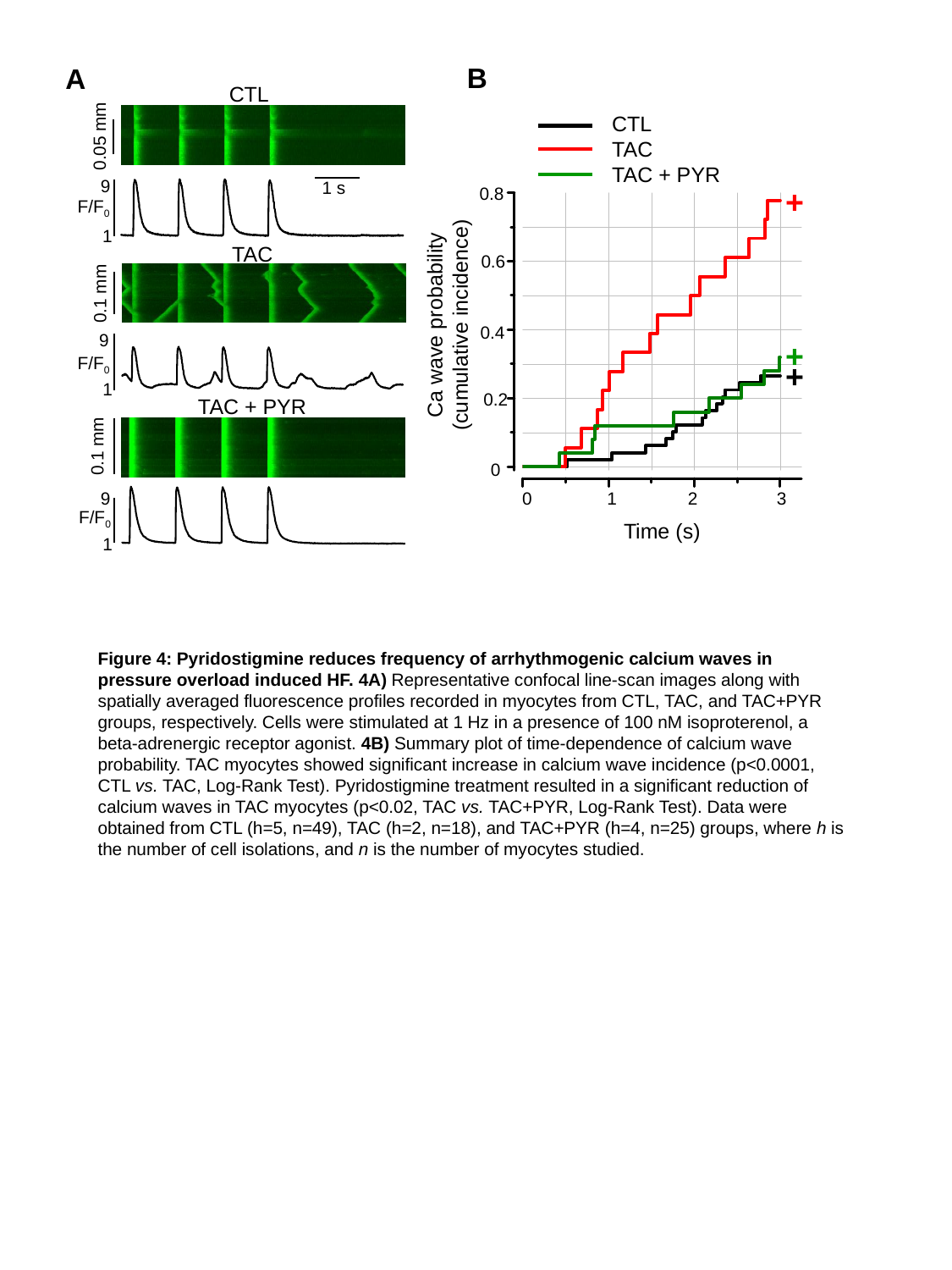

B
A
CTL
0.05 mm
9
1 s
F/F0
1
TAC
0.1 mm
9
F/F0
1
TAC + PYR
0.1 mm
9
F/F0
1
 CTL
 TAC
 TAC + PYR
+
0.8
0.6
Ca wave probability
(cumulative incidence)
0.4
+
+
0.2
0
0
1
2
3
Time (s)
Figure 4: Pyridostigmine reduces frequency of arrhythmogenic calcium waves in pressure overload induced HF. 4A) Representative confocal line-scan images along with spatially averaged fluorescence profiles recorded in myocytes from CTL, TAC, and TAC+PYR groups, respectively. Cells were stimulated at 1 Hz in a presence of 100 nM isoproterenol, a beta-adrenergic receptor agonist. 4B) Summary plot of time-dependence of calcium wave probability. TAC myocytes showed significant increase in calcium wave incidence (p<0.0001, CTL vs. TAC, Log-Rank Test). Pyridostigmine treatment resulted in a significant reduction of calcium waves in TAC myocytes (p<0.02, TAC vs. TAC+PYR, Log-Rank Test). Data were obtained from CTL (h=5, n=49), TAC (h=2, n=18), and TAC+PYR (h=4, n=25) groups, where h is the number of cell isolations, and n is the number of myocytes studied.

## Slide 5
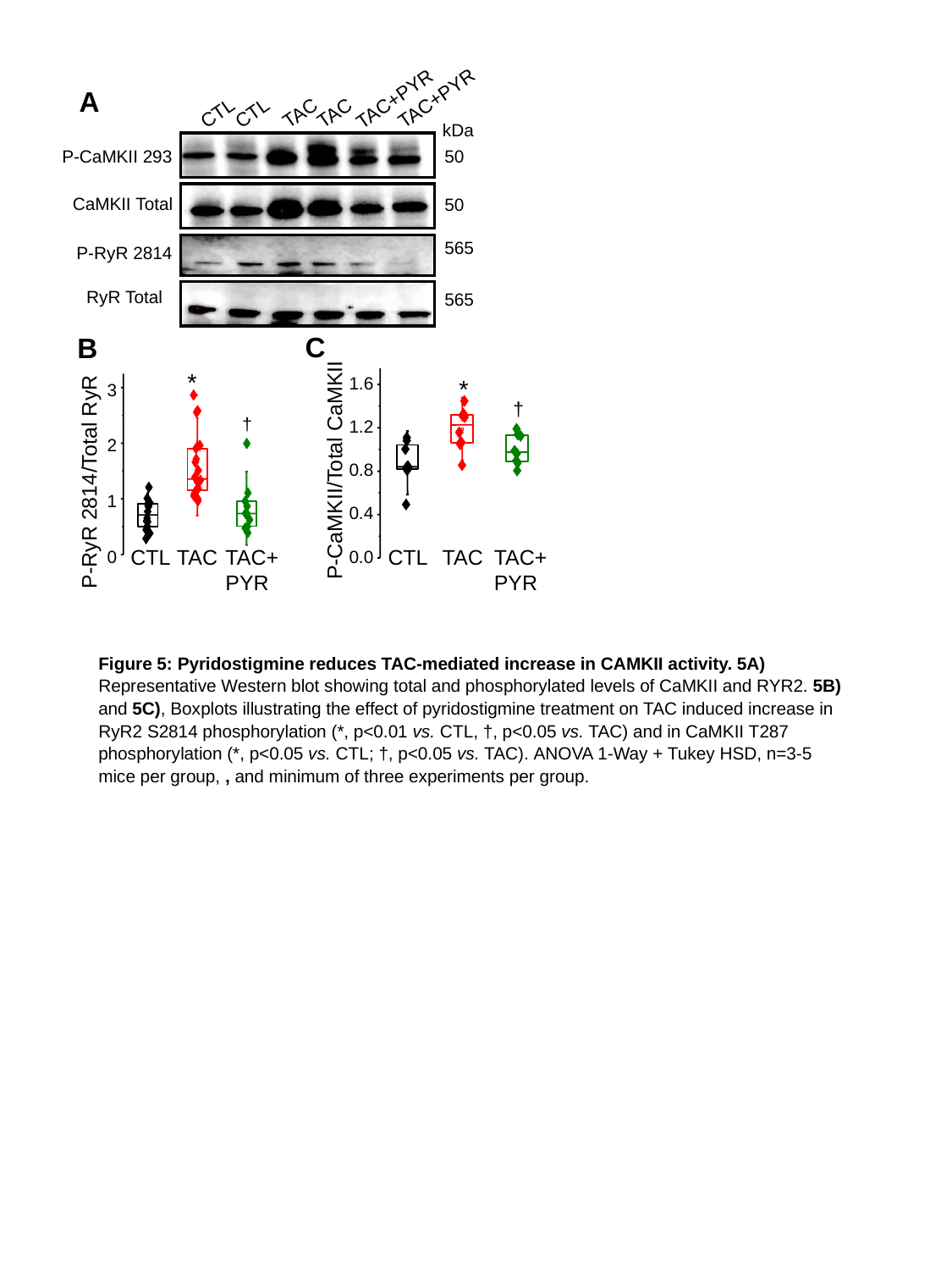

TAC+PYR
TAC+PYR
A
CTL
CTL
TAC
TAC
kDa
50
P-CaMKII 293
CaMKII Total
50
565
P-RyR 2814
RyR Total
565
C
B
*
3
†
2
1
0
1.6
*
†
1.2
0.8
0.4
0.0
P-CaMKII/Total CaMKII
P-RyR 2814/Total RyR
CTL
TAC
TAC+
PYR
CTL
TAC
TAC+
PYR
Figure 5: Pyridostigmine reduces TAC-mediated increase in CAMKII activity. 5A) Representative Western blot showing total and phosphorylated levels of CaMKII and RYR2. 5B) and 5C), Boxplots illustrating the effect of pyridostigmine treatment on TAC induced increase in RyR2 S2814 phosphorylation (*, p<0.01 vs. CTL, †, p<0.05 vs. TAC) and in CaMKII T287 phosphorylation (*, p<0.05 vs. CTL; †, p<0.05 vs. TAC). ANOVA 1-Way + Tukey HSD, n=3-5 mice per group, , and minimum of three experiments per group.

## Slide 6
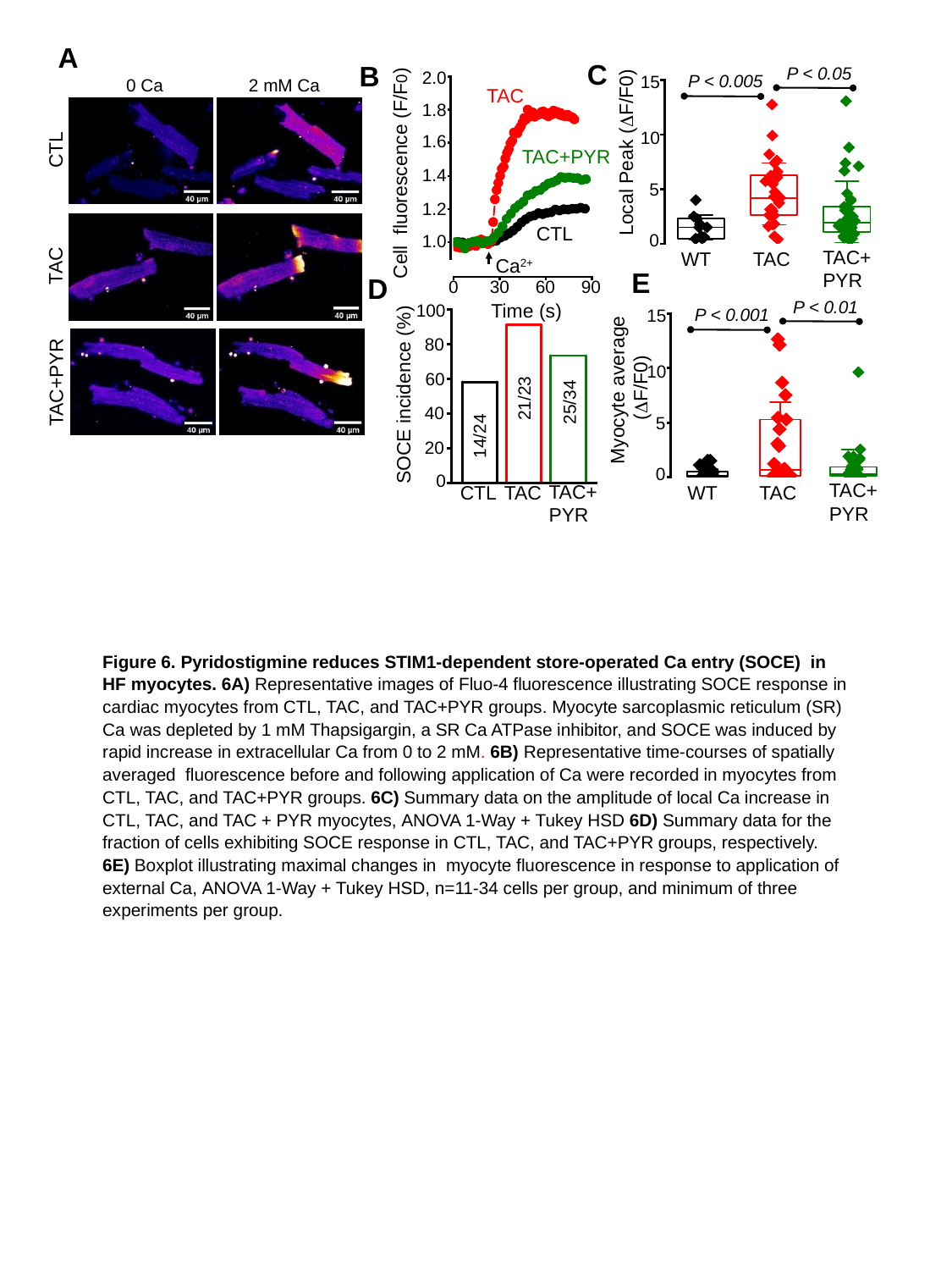

2.0
TAC
1.8
1.6
TAC+PYR
Cell fluorescence (F/F0)
1.4
1.2
CTL
1.0
0
30
60
90
Time (s)
Ca2+
A
P < 0.05
P < 0.005
15
10
Local Peak (DF/F0)
5
0
TAC+
PYR
WT
TAC
C
B
0 Ca
2 mM Ca
CTL
TAC
E
D
P < 0.01
P < 0.001
15
10
Myocyte average
(DF/F0)
5
0
TAC+
PYR
WT
TAC
100
80
60
TAC+PYR
SOCE incidence (%)
21/23
25/34
40
14/24
20
0
TAC+
PYR
TAC
CTL
Figure 6. Pyridostigmine reduces STIM1-dependent store-operated Ca entry (SOCE) in HF myocytes. 6A) Representative images of Fluo-4 fluorescence illustrating SOCE response in cardiac myocytes from CTL, TAC, and TAC+PYR groups. Myocyte sarcoplasmic reticulum (SR) Ca was depleted by 1 mM Thapsigargin, a SR Ca ATPase inhibitor, and SOCE was induced by rapid increase in extracellular Ca from 0 to 2 mM. 6B) Representative time-courses of spatially averaged fluorescence before and following application of Ca were recorded in myocytes from CTL, TAC, and TAC+PYR groups. 6C) Summary data on the amplitude of local Ca increase in CTL, TAC, and TAC + PYR myocytes, ANOVA 1-Way + Tukey HSD 6D) Summary data for the fraction of cells exhibiting SOCE response in CTL, TAC, and TAC+PYR groups, respectively. 6E) Boxplot illustrating maximal changes in myocyte fluorescence in response to application of external Ca, ANOVA 1-Way + Tukey HSD, n=11-34 cells per group, and minimum of three experiments per group.

## Slide 7
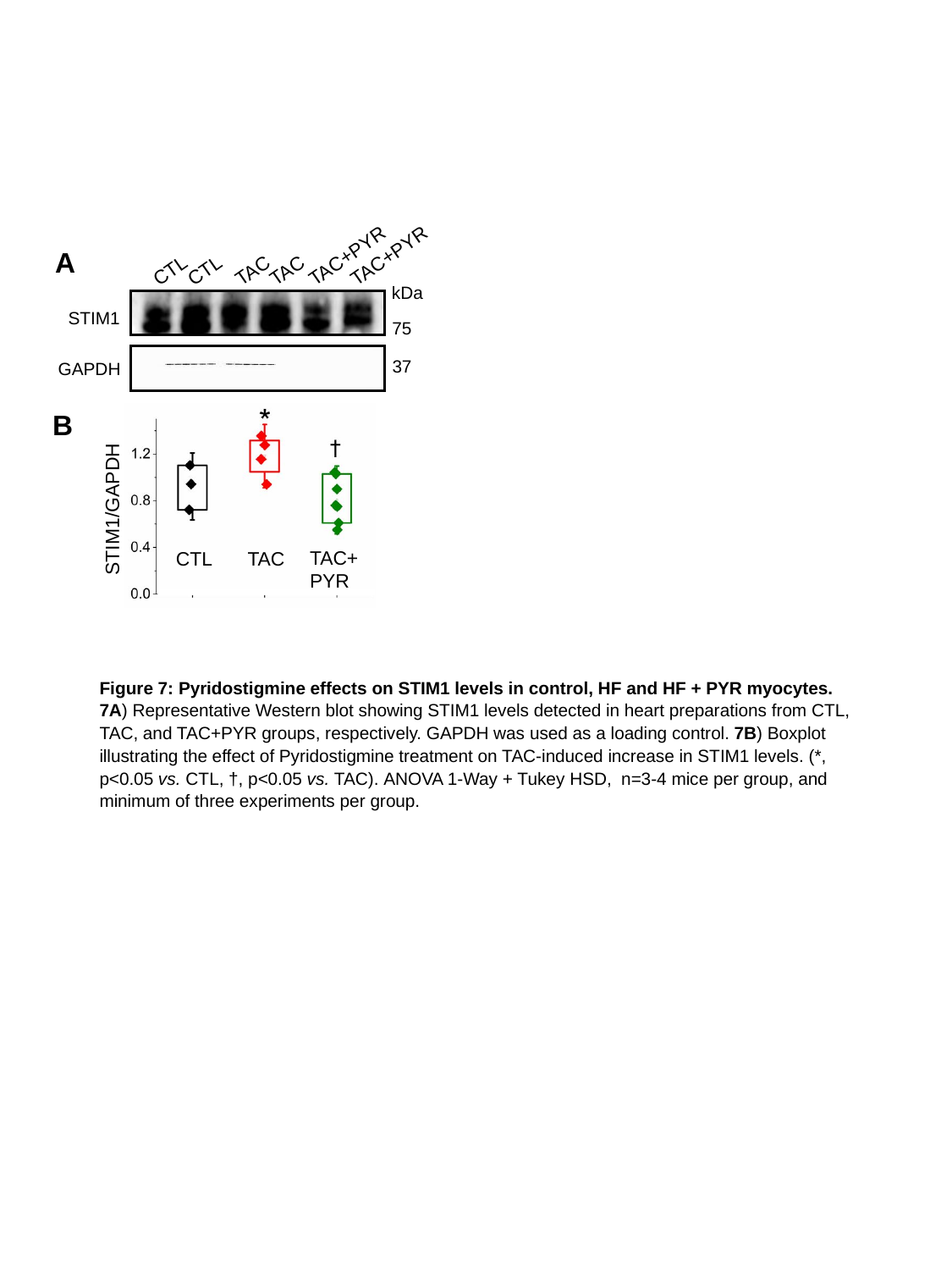

TAC+PYR
TAC+PYR
A
CTL
CTL
TAC
TAC
kDa
STIM1
75
37
GAPDH
B
STIM1/GAPDH
TAC+
PYR
TAC
CTL
Figure 7: Pyridostigmine effects on STIM1 levels in control, HF and HF + PYR myocytes. 7A) Representative Western blot showing STIM1 levels detected in heart preparations from CTL, TAC, and TAC+PYR groups, respectively. GAPDH was used as a loading control. 7B) Boxplot illustrating the effect of Pyridostigmine treatment on TAC-induced increase in STIM1 levels. (*, p<0.05 vs. CTL, †, p<0.05 vs. TAC). ANOVA 1-Way + Tukey HSD, n=3-4 mice per group, and minimum of three experiments per group.

## Slide 8
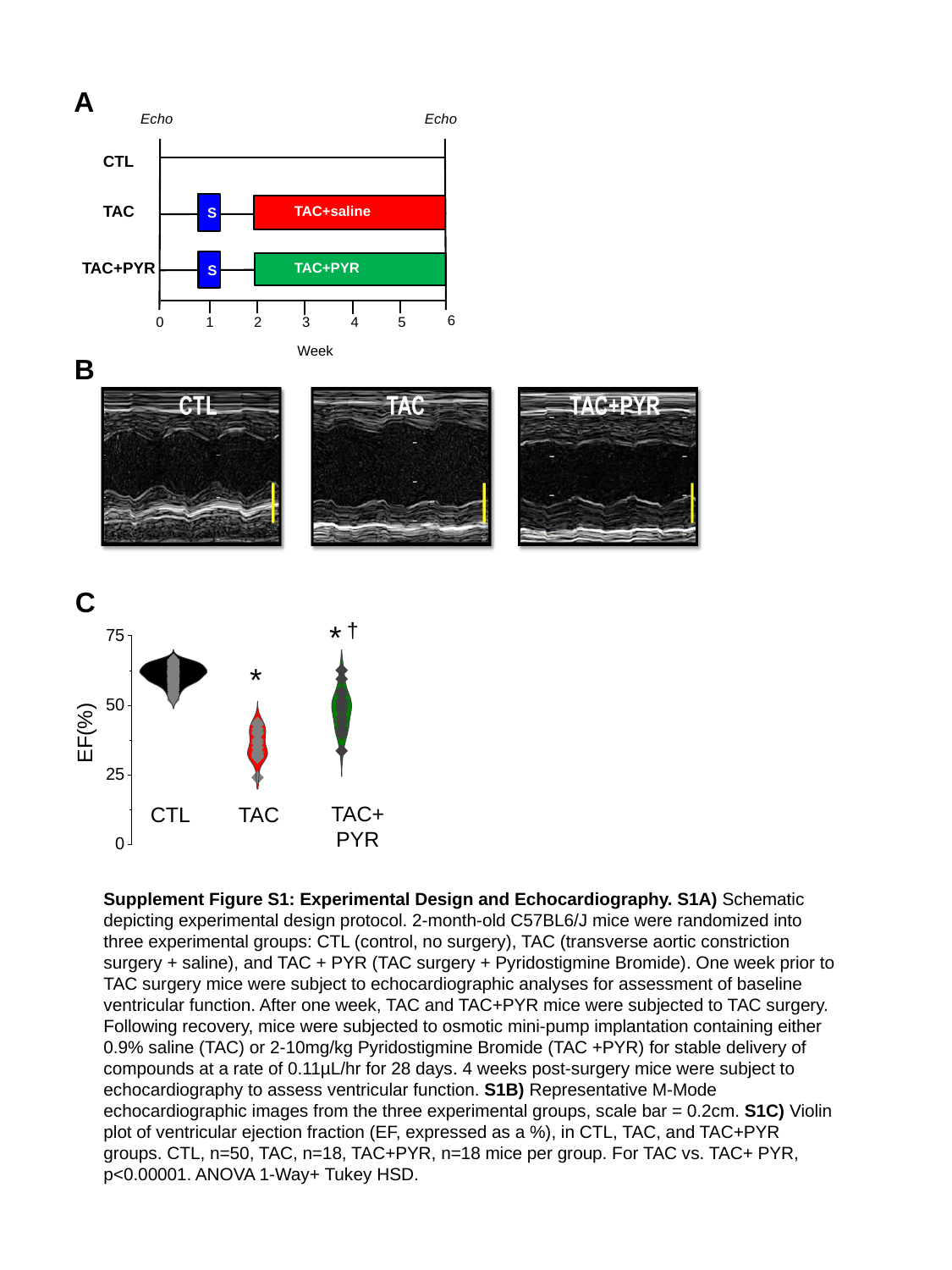

A
Echo
Echo
CTL
S
TAC
TAC+saline
S
TAC+PYR
TAC+PYR
6
0
1
2
3
4
5
Week
B
C
†
*
*
EF(%)
TAC+
PYR
CTL
TAC
Supplement Figure S1: Experimental Design and Echocardiography. S1A) Schematic depicting experimental design protocol. 2-month-old C57BL6/J mice were randomized into three experimental groups: CTL (control, no surgery), TAC (transverse aortic constriction surgery + saline), and TAC + PYR (TAC surgery + Pyridostigmine Bromide). One week prior to TAC surgery mice were subject to echocardiographic analyses for assessment of baseline ventricular function. After one week, TAC and TAC+PYR mice were subjected to TAC surgery. Following recovery, mice were subjected to osmotic mini-pump implantation containing either 0.9% saline (TAC) or 2-10mg/kg Pyridostigmine Bromide (TAC +PYR) for stable delivery of compounds at a rate of 0.11µL/hr for 28 days. 4 weeks post-surgery mice were subject to echocardiography to assess ventricular function. S1B) Representative M-Mode echocardiographic images from the three experimental groups, scale bar = 0.2cm. S1C) Violin plot of ventricular ejection fraction (EF, expressed as a %), in CTL, TAC, and TAC+PYR groups. CTL, n=50, TAC, n=18, TAC+PYR, n=18 mice per group. For TAC vs. TAC+ PYR, p<0.00001. ANOVA 1-Way+ Tukey HSD.

## Slide 9
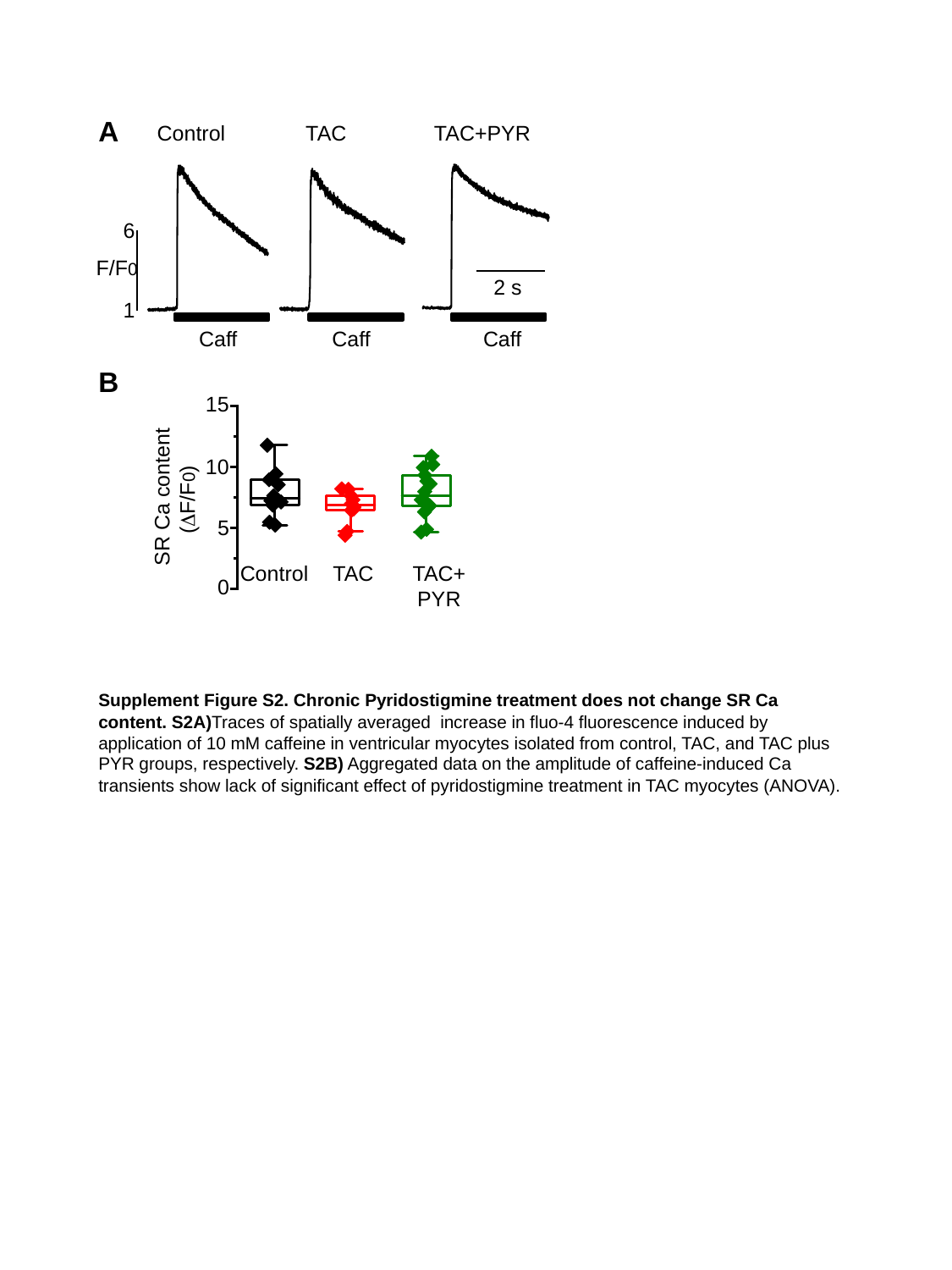

A
Control
TAC
TAC+PYR
6
F/F0
2 s
1
Caff
Caff
Caff
B
15
10
SR Ca content (DF/F0)
5
Control
TAC
TAC+
PYR
0
Supplement Figure S2. Chronic Pyridostigmine treatment does not change SR Ca content. S2A)Traces of spatially averaged increase in fluo-4 fluorescence induced by application of 10 mM caffeine in ventricular myocytes isolated from control, TAC, and TAC plus PYR groups, respectively. S2B) Aggregated data on the amplitude of caffeine-induced Ca transients show lack of significant effect of pyridostigmine treatment in TAC myocytes (ANOVA).

## Slide 10
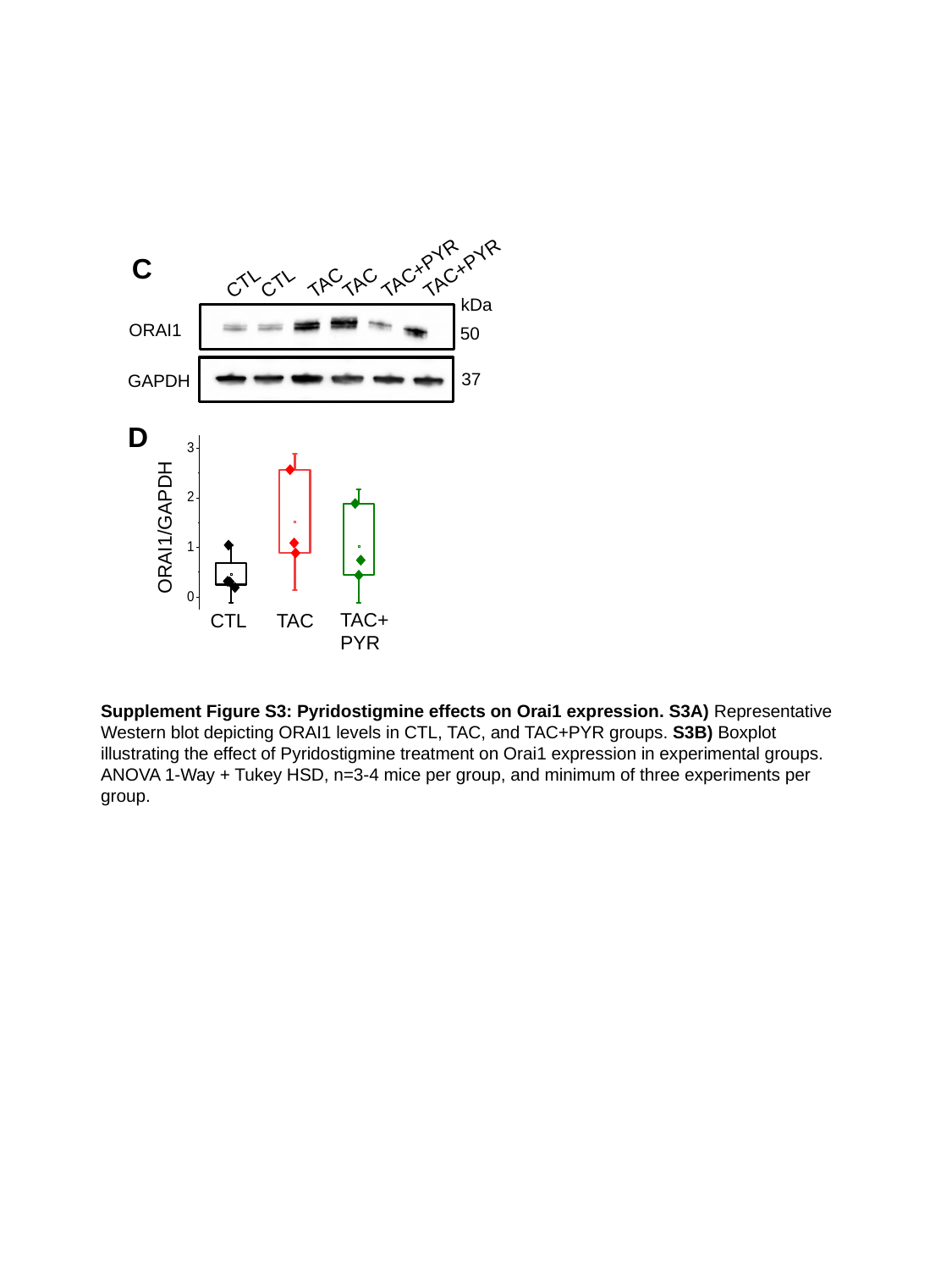

TAC+PYR
TAC+PYR
C
CTL
CTL
TAC
TAC
kDa
ORAI1
50
37
GAPDH
D
ORAI1/GAPDH
TAC+
PYR
TAC
CTL
Supplement Figure S3: Pyridostigmine effects on Orai1 expression. S3A) Representative Western blot depicting ORAI1 levels in CTL, TAC, and TAC+PYR groups. S3B) Boxplot illustrating the effect of Pyridostigmine treatment on Orai1 expression in experimental groups. ANOVA 1-Way + Tukey HSD, n=3-4 mice per group, and minimum of three experiments per group.

## Slide 11
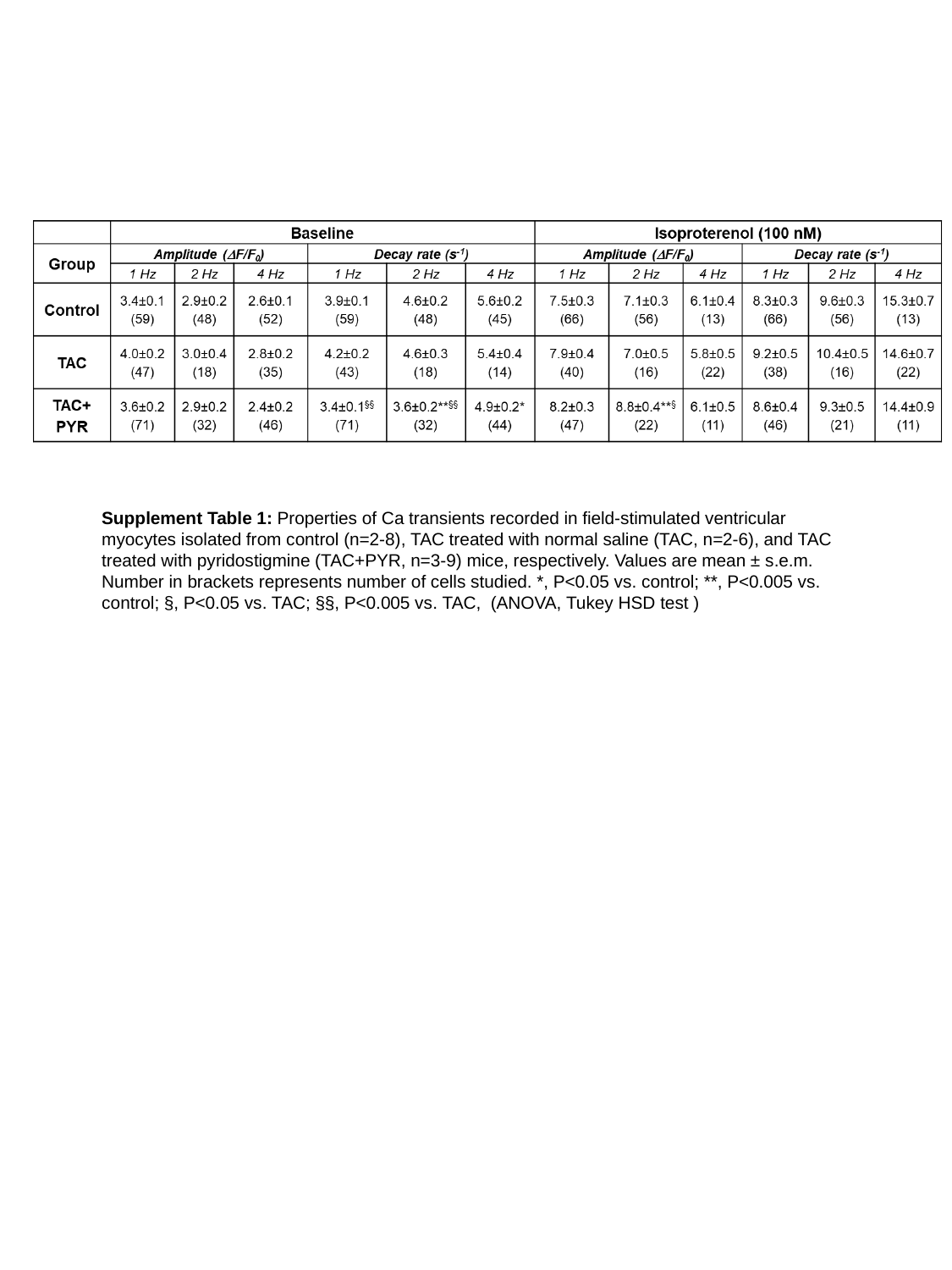

Supplement Table 1: Properties of Ca transients recorded in field-stimulated ventricular myocytes isolated from control (n=2-8), TAC treated with normal saline (TAC, n=2-6), and TAC treated with pyridostigmine (TAC+PYR, n=3-9) mice, respectively. Values are mean ± s.e.m. Number in brackets represents number of cells studied. *, P<0.05 vs. control; **, P<0.005 vs. control; §, P<0.05 vs. TAC; §§, P<0.005 vs. TAC, (ANOVA, Tukey HSD test )

## Slide 12
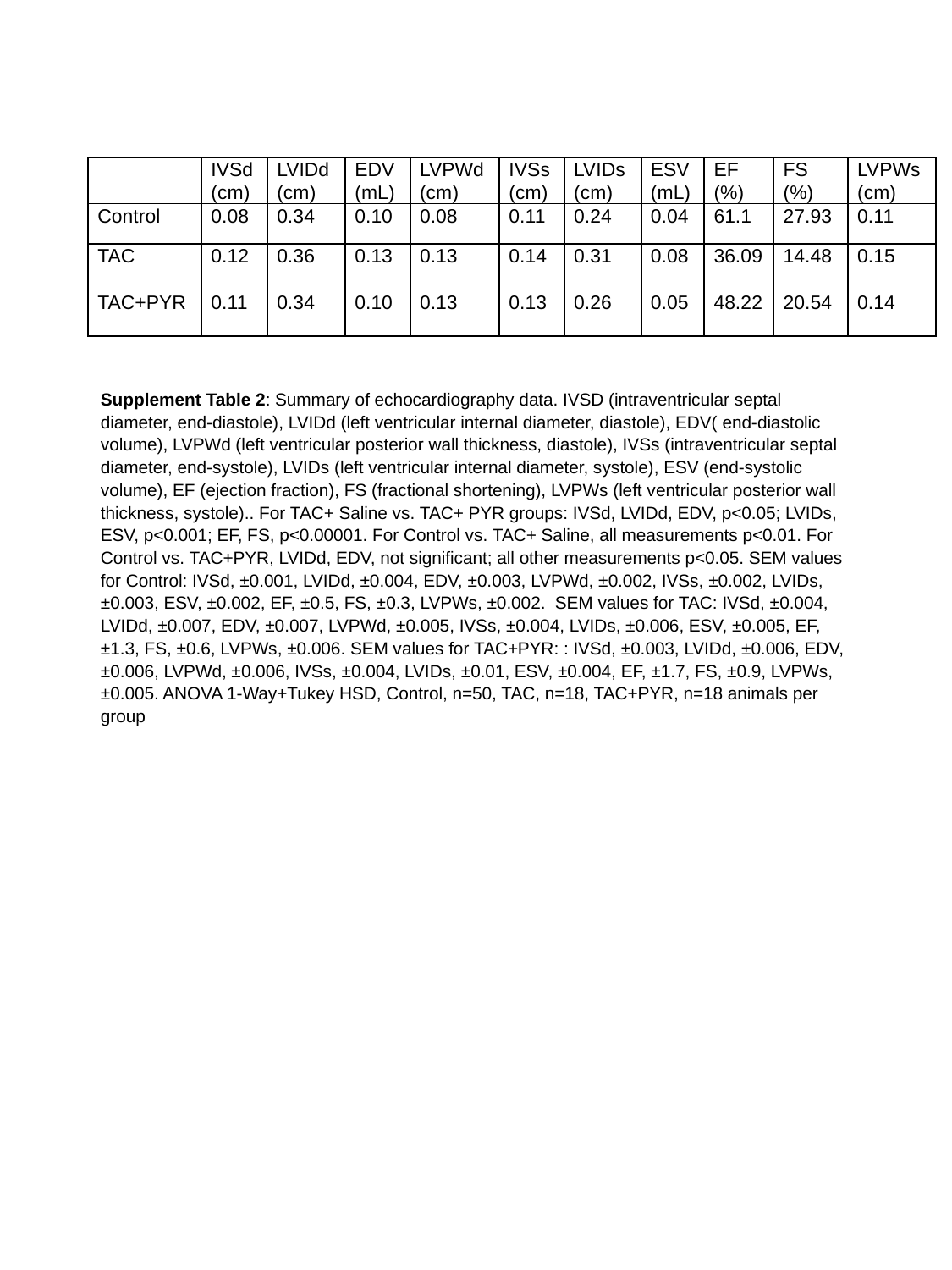

| | IVSd (cm) | LVIDd (cm) | EDV (mL) | LVPWd (cm) | IVSs (cm) | LVIDs (cm) | ESV (mL) | EF (%) | FS (%) | LVPWs (cm) |
| --- | --- | --- | --- | --- | --- | --- | --- | --- | --- | --- |
| Control | 0.08 | 0.34 | 0.10 | 0.08 | 0.11 | 0.24 | 0.04 | 61.1 | 27.93 | 0.11 |
| TAC | 0.12 | 0.36 | 0.13 | 0.13 | 0.14 | 0.31 | 0.08 | 36.09 | 14.48 | 0.15 |
| TAC+PYR | 0.11 | 0.34 | 0.10 | 0.13 | 0.13 | 0.26 | 0.05 | 48.22 | 20.54 | 0.14 |
Supplement Table 2: Summary of echocardiography data. IVSD (intraventricular septal diameter, end-diastole), LVIDd (left ventricular internal diameter, diastole), EDV( end-diastolic volume), LVPWd (left ventricular posterior wall thickness, diastole), IVSs (intraventricular septal diameter, end-systole), LVIDs (left ventricular internal diameter, systole), ESV (end-systolic volume), EF (ejection fraction), FS (fractional shortening), LVPWs (left ventricular posterior wall thickness, systole).. For TAC+ Saline vs. TAC+ PYR groups: IVSd, LVIDd, EDV, p<0.05; LVIDs, ESV, p<0.001; EF, FS, p<0.00001. For Control vs. TAC+ Saline, all measurements p<0.01. For Control vs. TAC+PYR, LVIDd, EDV, not significant; all other measurements p<0.05. SEM values for Control: IVSd, ±0.001, LVIDd, ±0.004, EDV, ±0.003, LVPWd, ±0.002, IVSs, ±0.002, LVIDs, ±0.003, ESV, ±0.002, EF, ±0.5, FS, ±0.3, LVPWs, ±0.002. SEM values for TAC: IVSd, ±0.004, LVIDd, ±0.007, EDV, ±0.007, LVPWd, ±0.005, IVSs, ±0.004, LVIDs, ±0.006, ESV, ±0.005, EF, ±1.3, FS, ±0.6, LVPWs, ±0.006. SEM values for TAC+PYR: : IVSd, ±0.003, LVIDd, ±0.006, EDV, ±0.006, LVPWd, ±0.006, IVSs, ±0.004, LVIDs, ±0.01, ESV, ±0.004, EF, ±1.7, FS, ±0.9, LVPWs, ±0.005. ANOVA 1-Way+Tukey HSD, Control, n=50, TAC, n=18, TAC+PYR, n=18 animals per group
